# Extruded and Enzyme-Fractionated Avocado (*Persea americana* Mill.) Seed Flour as an Ingredient for Frankfurter-Type Sausages: Technological, Physicochemical, and Sensory Implications

**DOI:** 10.3390/foods15091615

**Published:** 2026-05-06

**Authors:** Jesús Salvador Jaramillo-De la Garza, Esther Pérez-Carrillo, Carmen Hernández-Brenes, Dariana Graciela Rodríguez-Sánchez, Erick Heredia-Olea

**Affiliations:** 1 Escuela de Ingenieria y Ciencias, Tecnologico de Monterrey, Ave. Eugenio Garza Sada 2501, Monterrey 64700, NL, Mexico; sssalvador@tec.mx (J.S.J.-D.l.G.);; 2 Institute for Obesity Research, Tecnologico de Monterrey, Ave. Eugenio Garza Sada 2501, Monterrey 64700, NL, Mexico

**Keywords:** extrusion processing, enzyme-assisted fractionation, agro-industrial byproducts, avocado seed valorization, circular bioeconomy, frankfurter-type sausages, meat product reformulation, consumer acceptability

## Abstract

The valorization of agro-industrial byproducts has emerged as an important strategy to improve resource efficiency and promote circular food systems. This study evaluated avocado (*Persea americana* Mill.) seed as a functional ingredient for frankfurter-type sausages using extrusion followed by enzyme-assisted wet milling. Extrusion modified the techno-functional properties of avocado seed flour, increasing the water absorption index from 2.87 to 3.91 g/g while reducing the oil absorption index from 2.12 to 1.84 g/g. In addition, extrusion reduced the total acetogenin content by approximately 82.8% (11.99 to 2.07 mg/g), indicating a substantial reduction of these endogenous compounds. When incorporated at a concentration of 1% (*w*/*w*) to replace commercial soy fiber, avocado seed ingredients produced frankfurter-type sausages with low cooking losses (1.67–3.77%), stable water activity (0.979–0.990), and an acceptable instrumental hardness (1.01–1.41 N) over 35 days of refrigerated storage. Consumer sensory evaluation (*n* = 106) showed comparable or higher flavor and overall acceptability scores for sausages containing avocado seed flour relative to the control formulation. These findings demonstrate that extruded avocado seed flour can function as a viable upcycled ingredient for emulsified meat products, supporting circular bioeconomy approaches for the development of value-added foods of animal origin.

## 1. Introduction

The rapid expansion of global food production has intensified concerns regarding the generation and management of agro-industrial byproducts. Fruit and vegetable processing residues exhibit some of the highest loss and waste rates across the food supply chain. These residues, commonly consisting of peels, seeds, and other non-marketable fractions, represent an environmental challenge but also constitute a potential source of valuable components such as carbohydrates, dietary fiber, proteins, minerals, and bioactive compounds [1]. Consequently, increasing attention has been directed toward the valorization of these materials through processing strategies capable of converting underutilized plant residues into functional food ingredients [2,3].

Among fruit processing residues, avocado seed has recently emerged as a promising candidate for ingredient development. The growth in global avocado production, which reached approximately 8.98 million metric tons in 2022, has generated substantial quantities of peel and seed by-products. These residues can account for up to 40% of the processed fruit, representing more than 1.2 million tons of agro-industrial waste annually. The seed alone represents approximately 13–18% of the fruit weight [4,5]. Although commonly discarded, avocado seed contains significant amounts of starch, dietary fiber, protein, and phenolic compounds with notable antioxidant activity [5,6]. This composition has stimulated growing interest in its use as a functional food ingredient. However, most studies have focused on applications in cereal-based foods such as bakery products, snacks, and beverages [7,8], whereas its incorporation into meat products remains largely unexplored.

Despite its promising composition, the direct incorporation of native avocado seed flour into food formulations presents several limitations. High concentrations of phenolic compounds may contribute to bitterness, astringency, and undesirable color changes, which can negatively affect the sensory acceptability [4]. In addition, the native starch–fiber matrix limits the starch accessibility and solubility, often resulting in poor technological performance when it is used in food systems [6,9,10]. Safety considerations must also be addressed, as avocado tissues contain acetogenins such as persin, compounds associated with biological activity, including reported toxic effects in animal models, which may raise concerns regarding their direct use in food systems and therefore require processing strategies capable of reducing their concentration [5,7]. Therefore, processing strategies capable of modifying the structural organization of avocado seed flour while improving its techno-functional properties are required. Extrusion is widely recognized as an effective thermomechanical technology for modifying starch- and fiber-rich plant materials. The combined effects of heat, pressure, and shear stress during extrusion alters the structural organization of plant macromolecules improving the hydration behavior, viscosity, and overall techno-functional performance [11,12]. Enzymatic processing represents a complementary approach, as enzymes such as cellulases and hemicellulases can hydrolyze structural polysaccharides within plant cell walls, thereby enhancing the accessibility of fiber fractions. In addition, amylases can hydrolyze starch components, while proteases may disrupt the protein matrix, facilitating the release of embedded compounds and contributing to the modification of functional properties [2,13,14]. The combination of thermomechanical and enzymatic treatments has therefore gained attention as a strategy to enhance the functionality of plant-derived ingredients. These modifications are particularly relevant when plant-derived ingredients are incorporated into complex food matrices such as emulsified meat products. Plant dietary fibers have been widely reported to improve water-holding capacity, texture, and cooking yield in comminuted meat systems by reinforcing the stability of the protein–fat matrix [15]. In addition, phenolic-rich plant ingredients may contribute antioxidant functionality, helping to reduce lipid oxidation and improve color stability [16]. Consequently, the techno-functional properties and dietary fiber content of frankfurter-type sausages and similar products have been successfully enhanced by incorporating several fruit- and seed-derived by-products [17,18,19].

Despite all these advances mentioned, the application of avocado seed in emulsified meat products remains largely unexplored, particularly regarding structurally modified avocado seed ingredients obtained through combined thermomechanical and enzymatic treatments. Understanding how these ingredients interact with the protein–fat matrix of meat emulsions such as frankfurter-type sausages is essential to determine their potential as functional components in reformulated meat products. In this context, the techno-functional properties of avocado seed ingredients play a critical role in determining their performance within the meat matrix. Therefore, this study aimed to evaluate the incorporation of enzyme-assisted wet-milled avocado seed fractions derived from both non-extruded and extruded flours into frankfurter-type sausages, in order to assess the effect of extrusion prior to enzymatic fractionation on their physicochemical, technological, and sensory performance.

## 2. Materials and Methods

### 2.1. Materials

Analytical reagents, including sodium hydroxide, hydrochloric acid, nutrient agar (NA), eosin methylene blue (EMB) agar, and potato dextrose agar (PDA), were obtained from Sigma-Aldrich (St. Louis, MO, USA). Alcalase^®^ 2.4 L FG was supplied by Novozymes (Bagsværd, Denmark), and the total dietary fiber assay kit was purchased from Megazyme (Bray, Ireland). Organic solvents (toluene, chloroform, methanol, and hexane) were obtained from CTR Scientific (Monterrey, NL, Mexico), while HPLC-grade solvents were supplied by Tedia (Fairfield, OH, USA). Sulfuric acid was obtained from DEQ (San Nicolás de los Garza, NL, Mexico). Fresh avocado (*Persea americana* Mill., cv. Hass) seeds, generated as a by-product during guacamole preparation, were obtained from ripe fruits purchased at a local supermarket in Monterrey, NL, Mexico. Seeds were manually separated, washed with distilled water to remove adhering pulp residues, and transported for further processing. Analytical standards of avocado seed acetogenins (>97% purity) were purified in-house and identified by MS-TOF following previously reported methods [20], including representative compounds commonly found in avocado seed extracts.

### 2.2. Preparation and Modification of Avocado Seed Flours

**Figure 1:**
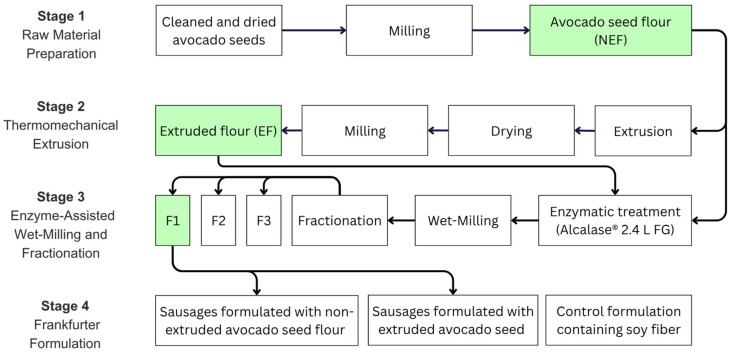
Schematic representation of the preparation and modification of avocado seed flours, including extrusion and enzyme-assisted wet-milling fractionation, and their subsequent incorporation into frankfurter-type sausage formulations. NEF: non-extruded avocado seed flour; EF: extruded avocado seed flour; F1–F3: fractions obtained after enzyme-assisted wet milling of both NEF and EF.

Avocado seeds were subjected to a sequential processing approach to obtain non-extruded flour (NEF), extruded flour (EF), and fractions derived from enzyme-assisted wet milling, as illustrated in Figure 1. Cleaned avocado seeds were processed to obtain a native, non-extruded flour (NEF). Approximately 10 kg of avocado seeds, with an initial moisture content of 5.15% (wet basis), were first reduced in size by coarse grinding using a meat grinder (model M-12-FS, Torrey, Guadalupe, NL, Mexico). The resulting coarse material was then dried in a convection oven (Electrolux EOG Gas single oven X 601, Stockholm, Sweden) at 55 °C for 16 h to further reduce moisture content and facilitate subsequent milling. Dried samples were then milled in a knife mill (Wiley Mill, Standard Model No. 3, Philadelphia, PA, USA) equipped with a 1 mm screen to obtain a fine flour. The resulting NEF was vacuum-sealed and stored at 25 ± 2 °C until further use. Flour preparation was carried out in a single batch, and the NEF obtained was used as the starting material for extrusion processing and enzyme-assisted wet-milling treatments described below.

#### 2.2.1. Extrusion Processing of Avocado Seed Flour

The non-extruded avocado seed flour (NEF) was subjected to thermomechanical processing by extrusion to obtain extruded flour (EF). Extrusion was carried out using a co-rotating twin-screw extruder (BTSM-30, Bühler AG, Uzwil, Switzerland) equipped with 600 mm length screws and 30 mm diameter (L/D = 20). A high-shear screw configuration [21], was employed to promote intensive shear stress; operating conditions were adapted from cereal-based extrusion systems [22] and maintained constant throughout the process. A temperature profile was applied along the barrel, reaching a maximum setpoint of 130 °C in the final zone, while the product temperature at the die was approximately 81 °C. The solids and water feed rates were set at 3.5 kg/h and 6.0 kg/h, respectively, according to the equipment settings, while the screw speed was set at 500 rpm. Extrusion was performed using a single-circular orifice die of 4 mm diameter. Under these conditions, the specific mechanical energy (SME) reached 114.82 ± 1.71 Wh/kg, and the average torque was 24.23 ± 0.40% (58.90 ± 0.85 N·m), calculated as the mean of three independent extrusion runs. After exiting the die, extrudates were subjected to drying in a convection oven (Electrolux EOG Gas single oven X 601, Stockholm, Sweden) at 55 °C for 16 h, followed by equilibration at 25 ± 2 °C prior to size reduction. Milling was performed using a knife mill (Wiley Mill, Model No. 3, Philadelphia, PA, USA) equipped with a 1 mm screen to obtain the final flour. The resulting extruded flour (EF) was vacuum-packaged and stored at 25 ± 2 °C until further use. Extrusion processing was carried out on three separate days under identical operating conditions, and each production run was considered an independent batch. A total of three independent batches were produced. 

#### 2.2.2. Enzyme-Assisted Wet Milling and Fractionation

Enzyme-assisted wet milling was applied to both non-extruded (NEF) and extruded (EF) avocado seed flours. Briefly, 320 g of flour was suspended in 1.6 L of distilled water and processed in a 2 L bioreactor (Biostat A Plus, Sartorius BBI Systems, Göttingen, Germany). A commercial protease preparation (Alcalase® 2.4 L FG, Novozymes, Bagsværd, Denmark) was added at an enzyme-to-substrate ratio of 1 mL per 50 g of flour (1:50, *v*/*w*, enzyme:substrate). The reaction mixture was incubated for 2 h at pH 8.0 and 40 °C under continuous agitation (150 rpm), following the conditions described by Xiao et al. [23]. After enzymatic hydrolysis, the slurry was subjected to wet milling by blending for 1 min using a commercial blender (Oster, Boca Raton, FL, USA) and subsequently sieved through an 80-mesh sieve. The material retained on the sieve (fraction 1, F1) was re-blended for 30 s and sieved again to improve separation efficiency. All filtrates were pooled and centrifuged at 3000 rpm for 5 min (≈805× *g*). The resulting pellet was designated as fraction 2 (F2), while the supernatant was collected as fraction 3 (F3). All fractions were dried in a convection oven (Electrolux EOG Gas single oven X 601, Stockholm, Sweden) at 55 °C until constant weight was reached. The average drying time was 19.7 ± 3.6 h, mainly influenced by the initial mass of the samples. Based on preliminary technological screening trials and its favorable techno-functional properties for emulsified meat systems, fraction 1 (F1) was selected for incorporation into frankfurter-type sausages. The enzyme-assisted wet milling and fractionation procedures were conducted in multiple independent batches (six independent batches for NEF and four for EF), and results are reported as mean ± SD across these replicates.

### 2.3. Analysis of Avocado Seed Flours and Fractions

#### 2.3.1. Chemical Composition

The chemical composition of avocado seed ingredients and frankfurter-type sausages was determined. Analyses were performed on non-extruded flour (NEF), extruded flour (EF), enzyme-assisted wet-milled fractions from both flours, and the corresponding sausage formulations. The moisture content, total dietary fiber (TDF), insoluble dietary fiber (IDF), and soluble dietary fiber (SDF) were determined according to AACC 32-05.01 and 32-07.01 methods [24]. Ash content was quantified by dry incineration of approximately 1 g of the sample in a muffle furnace (Barnstead Thermolyne, Model FA7915, Dubuque, IA, USA) at 550 °C for 5 h, according to AOAC Method 923.03 [25]. The crude fat content was determined by Soxhlet extraction using petroleum ether as extraction solvent (AOAC Method 920.85). Crude protein was determined by the Kjeldahl method according to AOAC Method 978.02. Nitrogen values were converted to protein using a conventional nitrogen-to-protein conversion factor of 6.25. The carbohydrate content was calculated by difference on a dry weight basis, as described by [26], as 100 minus the sum of moisture, protein, fat, ash, and total dietary fiber. All determinations were performed in triplicate, except for dietary fiber analyses, which were performed in quadruplicate. The replicates corresponded to the technical replicates obtained from the homogenized subsamples of each flour type and sausage formulation.

#### 2.3.2. Acetogenin Content

The content of acetogenins was determined in non-extruded avocado seed flour (NEF) and extruded flour (EF) to evaluate the effect of extrusion processing on these compounds. Acetogenins were extracted following the methodology reported by [27], with minor modifications. Briefly, 2 g of sample were mixed with 15 mL of acetone and homogenized using a high-shear homogenizer (Ultra-Turrax T25, IKA-Werke, Staufen im Breisgau, Germany) at 16,000 rpm (≈22,000× *g*) for 3 min. The homogenate was subsequently sonicated for 1 min in an ultrasonic bath (Model 50T, VWR, Radnor, PA, USA) and agitated for 15 min at 25 ± 2 °C using a multi-shaker (Lab-Line Incubator-Shaker, Thermo Fisher Scientific, Chicago, IL, USA). After extraction, the samples were centrifuged at 10,564 rpm (≈10,000× *g*) for 10 min. The resulting organic supernatant was collected and evaporated up to dryness under a gentle nitrogen stream. The dried extract was reconstituted with 2 mL of hexane and 2 mL of distilled water, vortexed, and centrifuged at 5000× *g* to promote phase separation. The organic phase was recovered and used for acetogenin quantification. The acetogenins were identified and quantified using an HPLC system (Agilent 1260 Infinity, Agilent Technologies, Santa Clara, CA, USA) equipped with a photodiode array (PDA) detector (model G4212B, Agilent Technologies). Chromatographic separation and detection conditions were based on the method previously described by [20]. The PDA detector was set at 208 nm for the quantification of persin and persenone B, and at 220 nm for the remaining acetogenins. The compounds quantification was carried out using the external standard method, employing five-point calibration curves constructed individually for each available purified acetogenin standard. The acetogenin concentrations were calculated from peak areas and expressed as mg/g dry weight. All extractions were carried out in triplicate, and each extract was analyzed chromatographically in technical duplicate.

#### 2.3.3. Antioxidant Activity

Antioxidant activity of NEF and EF avocado seed flours was determined using the DPPH radical scavenging assay according to the method of Brand-Williams [28], with minor modifications. The reaction was initiated by combining sample extracts with a freshly prepared DPPH solution, followed by incubation at 37 °C for 60 min under controlled conditions. Absorbance was then recorded at 515 nm using a UV–Vis microplate spectrophotometer (Fluostar Omega, BMG LABTECH, Ortenberg, Germany). Results were expressed as the percentage of DPPH radical inhibition relative to a control without sample extract. Measurements were conducted in triplicate using homogenized samples from each treatment.

#### 2.3.4. Water and Oil Absorption Indices (WAI and OAI)

The water absorption index (WAI) and oil absorption index (OAI) of avocado seed flours were determined according to the method described by Serna-Saldívar [29]. Analyses were performed on NEF, EF, and the enzyme-assisted wet-milled fractions. All measurements were performed in triplicate using homogenized samples. The indices were calculated using the following equations:

WAI (g/g) = Weight of hydrated sediment (g)/Dry weight of sample (g)(1)

OAI (g/g) = Weight of oil-retained sediment (g)/Dry weight of sample (g)(2)

#### 2.3.5. Emulsifying Capacity and Emulsion Stability

The emulsifying capacity (EC) and emulsion stability (ES) of avocado seed flours were determined following the method described by de la Rosa-Millán [30] with minor modifications. Briefly, 1 g of sample was dispersed in 25 mL of distilled water and homogenized using an Ultra-Turrax T25 homogenizer (IKA-Werke, Staufen im Breisgau, Germany) at 10,000 rpm for 1 min at approximately 25 °C. The pH of the dispersion was then adjusted to 7.0 using either 0.1 M NaOH or 0.1 M HCl. Subsequently, 25 mL of soybean oil were added to the dispersion, and the mixture was homogenized again at 10,000× *g* (≈10,564 rpm) for 1 min to promote the formation of an oil–water emulsion. The resulting emulsion was centrifuged at 1300× *g* for 5 min to promote phase separation. The emulsifying capacity was expressed as the percentage of the emulsified layer relative to the total height of the sample and was calculated according to Equation (3). All measurements were performed in triplicate.

EC (%) = (height of emulsified layer (mm)/total height of mixture (mm)) × 100(3)

To evaluate emulsion stability, the previously formed emulsions were heated in a water bath at 80 °C for 30 min and subsequently centrifuged again at 1300× *g* for 5 min. Emulsion stability was expressed as the percentage of the remaining emulsified layer relative to the initial height of the emulsion and was calculated according to Equation (4).

ES (%) = (height of remaining emulsion layer (mm)/initial height of emulsified layer (mm)) × 100(4)

#### 2.3.6. Pasting Properties (RVA)

The pasting behavior of avocado seed flours was evaluated using a Rapid Visco Analyzer (RVA model 1170, Newport Scientific, Warriewood, Australia). Measurements were conducted on NEF, EF, and the enzyme-assisted wet-milled fractions obtained from the non-extruded flour (NEF F1 and NEF F2). Samples (2.5 g, adjusted to 14% moisture basis) were subjected to a standard heating and cooling program consisting of an initial holding period at 50 °C for 1.5 min, heating to 95 °C over 3.5 min, holding at 95 °C for 2.5 min, cooling to 50 °C over 3.5 min, and a final holding step at 50 °C for 2.0 min. Viscosity parameters recorded included peak viscosity, breakdown, final viscosity, and setback. All analyses were performed in triplicate, and results are reported as mean ± SD.

#### 2.3.7. In Vitro Protein Digestibility

In vitro protein digestibility was evaluated using a multi-enzymatic pH-drop method based on the procedure described by Hsu [31], as subsequently applied in studies evaluating thermally processed plant ingredients [32]. Casein was used as protein of reference for the assay. Analyses were performed on non-extruded flour (NEF), extruded flour (EF), and the enzyme-assisted wet-milled fractions. Sample suspensions were prepared to obtain a protein concentration of 6.25 mg/mL in distilled water and adjusted to pH 8.0. The reaction mixtures were placed in a temperature-controlled water bath (Memmert, Schwabach, Germany) at 37 °C under continuous agitation. Enzymatic digestion was initiated by adding 5 mL of a freshly prepared multienzyme solution containing trypsin (1.6 mg/mL), chymotrypsin (3.1 mg/mL), and *Streptomyces griseus* protease (1.3 mg/mL), all adjusted to pH 8.0. The pH of each reaction mixture was recorded exactly 10 min after enzyme addition. In vitro protein digestibility (%) was calculated according to Equation (5) proposed by Hsu [31].

(5) $$\mathrm{In}\;\mathrm{vitro}\;\mathrm{protein}\;\mathrm{digestibility}\;(\%)=210.46-18.1\times {\mathrm{pH}}_{10\;\min}$$

In addition, relative protein digestibility was calculated using casein as the reference protein, following the approach described by Lazo [33], as shown in Equation (6):

(6) $$\mathrm{Relative}\;\mathrm{protein}\;\mathrm{digestibility}\;(\%)\;=\frac{\Delta {\mathrm{pH}}_{\mathrm{sample}}}{\Delta {\mathrm{pH}}_{\mathrm{casein}}}\times 100$$

All analyses were performed in triplicate as independent experimental replicates, and results are expressed as mean ± SD.

### 2.4. Frankfurter Formulation and Processing

#### 2.4.1. Experimental Formulations

Three frankfurter-type sausage formulations were prepared to evaluate the effect of incorporating ingredients derived from avocado seed flour. A control formulation (CNF) was produced using commercial soy fiber at an inclusion level of 1% (*w*/*w*). Two experimental formulations were prepared by completely replacing soy fiber with avocado seed flour ingredients: sausages formulated with non-extruded avocado seed flour (SNF) and sausages formulated with extruded avocado seed flour (SEF). All formulations were prepared using the same base recipe and identical processing conditions, differing only in the type of dietary fiber ingredient incorporated. To minimize variability, all formulations were produced using the same batch of raw materials and under identical processing conditions. The inclusion level (1%, *w*/*w*) was selected based on the typical use of commercial soy fiber in frankfurter-type sausage formulations and was applied consistently across treatments to allow direct comparison of ingredient functionality under equivalent conditions. This level is also consistent with commonly reported incorporation ranges for fiber ingredients in emulsified meat systems, where relatively low inclusion levels are required to maintain product stability and sensory acceptability. Based on preliminary technological screening trials and its favorable techno-functional properties for emulsified meat systems, fraction 1 (F1) was selected as the avocado seed ingredient used to replace soy fiber in frankfurter-type sausages, while fractions F2 and F3 were not considered for product formulation. The detailed composition of each formulation is presented in Table 1.

#### 2.4.2. Frankfurter Processing

Frankfurter processing was carried out at the pilot-plant facilities of a commercial meat processor, following standard industrial procedures for emulsified sausages. Turkey meat (boneless and skinless) was used as the primary raw material. All formulations were prepared using the same batch of raw materials and identical processing conditions to ensure consistency across treatments. Meat batters were prepared under controlled temperature conditions to prevent excessive heating during emulsification and to maintain emulsion stability, and subsequently processed as follows:

1. Meat emulsification: meat, water, salt, curing agents, and functional ingredients were mixed in a bowl cutter (JR65-G220, Supor Co., Ltd., Hangzhou, Zhejiang, China). The temperature of the batter was controlled during mixing by applying standard industrial practices for emulsified meat systems, including the use of chilled ingredients and control of processing time, in order to limit heat generation and maintain adequate emulsion formation.
2. Stuffing: the resulting meat batters were stuffed into 26 mm cellulose casings and manually linked.
3. Thermal processing: sausages were cooked at 80 °C for 25 min, followed by heating at 85 °C until an internal temperature of 78 °C was reached.
4. Cooling: cooked sausages were rapidly cooled to approximately 8 °C.
5. Packaging and storage: sausages were manually peeled, vacuum-packaged, and stored under refrigerated conditions (4 °C) until further analyses.

Frankfurter production was performed once per formulation at pilot-plant scale. The resulting batch for each formulation was used to obtain multiple independent sample units for physicochemical and sensory analyses. Individual sausages obtained from each batch were used as analytical units for physicochemical measurements.

### 2.5. Analysis of Frankfurter-Type Sausages

#### 2.5.1. Instrumental Color Measurement

Instrumental color measurements of frankfurter samples were performed using a portable colorimeter (Konica Minolta CM-600D, Osaka, Japan) operating under the CIE L*a*b* color space. The parameters recorded were lightness (L*), redness–greenness (a*), and yellowness–blueness (b*). Measurements were conducted using illuminant D65 and a 10° standard observer. Samples were measured at 25 ± 2 °C on the surface of sliced frankfurters. For each sample, nine readings were obtained at different positions and averaged. Chroma (C*) and hue angle (h°) were calculated from the L*a*b* coordinates according to Equations (7) and (8), respectively, following CIE recommendations [34].

(7) $$C*=\sqrt{\left(a*{)}^{2}+(b*{)}^{2}\right.}$$

(8) $$h^{\circ }=arctan\left(\frac{b*}{a*}\right)\times \frac{180}{\pi }$$

#### 2.5.2. pH and Water Activity (Aw)

The pH and water activity (Aw) of frankfurter samples were evaluated during refrigerated storage to monitor physicochemical stability over time. Measurements were carried out on day 0 (after processing) and after 7, 14, 21, 28, and 35 days of storage at 4 ± 1 °C. For pH determination, approximately 10 g of each frankfurter sample were homogenized with 90 mL of distilled water (1:10, *w*/*v*) using a laboratory blender. The pH of the homogenate was measured at 25 ± 2 °C using a calibrated pH meter (Symphony H30PCO, VWR, Radnor, PA, USA). Water activity (Aw) was measured at 25 °C using a dew-point water activity meter (Novasina TH-500, Lachen, Switzerland). Samples were placed directly in the measurement chamber without prior dilution and allowed to equilibrate before recording. All measurements were performed in triplicate for each formulation (CNF, SNF, and SEF) and storage time. Results are reported as mean ± SD.

#### 2.5.3. Cooking Loss

Cooking loss of frankfurter samples was determined to evaluate weight loss during thermal processing. For each formulation (CNF, SNF, and SEF), individual sausages were weighed before cooking (raw weight) and immediately after thermal processing and cooling (cooked weight). Cooking loss was calculated on a wet basis, using the total sample weight without moisture correction, as the percentage difference between raw and cooked weights according to Equation (9):

(9) $$\mathrm{Cooking}\;\mathrm{loss}\;(\%)\;=\frac{W_{\mathrm{raw}}-W_{\mathrm{cooked}}}{W_{\mathrm{raw}}}\times 100$$

where $W_{\mathrm{raw}}$ corresponds to the weight of the sausage before cooking and $W_{\mathrm{cooked}}$ corresponds to the weight after cooking and cooling. Measurements were performed in triplicate for each formulation, and results are expressed as mean ± SD.

#### 2.5.4. Instrumental Texture Analysis

The instrumental texture properties of frankfurter samples were evaluated during refrigerated storage to monitor changes in mechanical resistance over time. Measurements were conducted on day 0 (after processing) and after 7, 14, 21, 28, and 35 days of storage at 4 ± 1 °C. Texture analysis was performed using a texture analyzer (TVT 6700, Perten Instruments, Hägersten, Sweden). Samples were subjected to a single-cycle compression test under controlled conditions. Prior to analysis, frankfurter samples were equilibrated to 25 ± 2 °C and positioned centrally beneath the probe to ensure uniform axial compression. The equipment was fitted with an aluminum cylindrical probe of 45 mm diameter, and test parameters included a crosshead speed of 1 mm/s and a compression distance of 30 mm. The maximum force recorded during the first compression cycle (Force 1) was defined as instrumental hardness (N), representing the resistance of the frankfurter matrix to deformation. For each formulation (CNF, SNF, and SEF), 7–9 independent samples were analyzed at each storage time. Results are expressed as mean ± SD.

#### 2.5.5. Consumer Sensory Evaluation

Prior to sensory evaluation, all formulations were verified to comply with microbiological safety criteria for ready-to-eat meat products, as no total coliforms, aerobic mesophilic bacteria, or molds and yeasts were detected at day 0. Additionally, the products met the microbiological criteria for ready-to-eat meat products, indicating their suitability for consumer sensory evaluation. A consumer sensory evaluation was conducted to assess the acceptability of frankfurter-type sausages formulated with avocado seed ingredients. The evaluation compared a control formulation (CNF) with frankfurters containing 1% non-extruded avocado seed flour (SNF) or 1% extruded avocado seed flour (SEF). The consumer panel consisted of 106 untrained participants (students and staff from Tecnologico de Monterrey, Monterrey, Nuevo Leon, Mexico) who reported consuming sausages at least once per week. The participant’s mean age was 23 years. All individuals provided written informed consent, and the protocol was approved by the Institutional Ethics Committee (CSER-DBT-251022). Samples were prepared as uniform portions (~2 cm height), coded with random three-digit numbers, and presented in randomized order. Water and unsalted crackers were provided for palate cleansing between samples. Sensory attributes (flavor, texture, freshness, and overall acceptability) were evaluated using a 9-point hedonic scale.

### 2.6. Statistical Analysis

All measurements were performed using the number of independent replicates specified in each analytical section. Technical replicates were included where specified in each analytical section. Prior to statistical analysis, data were evaluated for normality, independence, and homogeneity of variances using the Shapiro–Wilk and Levene’s tests, respectively. For the characterization of avocado seed flours and their wet-milling fractions, comparisons between NEF and EF samples were performed using Student’s *t*-test when homogeneity of variances was satisfied, or Welch’s *t*-test when this assumption was not met. Due to the compositional heterogeneity of the fractions obtained through enzyme-assisted wet milling, these were not considered as equivalent treatments for direct statistical comparison, and differences among fractions were interpreted descriptively. Comparisons among multiple treatments were analyzed using one-way ANOVA, followed by Tukey’s honestly significant difference (HSD) post hoc test when significant differences were detected; when homogeneity of variances was not satisfied, Welch’s ANOVA was applied. For frankfurter formulations evaluated during refrigerated storage, data were analyzed using a two-way analysis of variance within a General Linear Model (GLM), considering treatment (T: CNF, SNF, SEF) and storage time (S: 0–35 days) as fixed factors, as well as their interaction (T × S). When significant effects were detected, mean comparisons among treatments at each storage time were performed using Tukey’s HSD test. When significant interaction effects (T × S) were observed, the interpretation of results was based on the combined influence of treatment and storage time. When appropriate, mean comparisons among treatments were performed within each storage time to facilitate interpretation. Consumer sensory data from 106 consumers were evaluated for normality using the Kolmogorov–Smirnov test, which indicated non-normal distribution of the hedonic scores. Therefore, sensory attributes were analyzed using the non-parametric Kruskal–Wallis test, followed by pairwise Mann–Whitney U tests with Bonferroni correction to control for multiple comparisons. All statistical analyses were conducted using Minitab version 22.2.1 (Minitab Inc., State College, PA, USA). Differences were considered statistically significant using an α = 0.05 unless otherwise specified. Results are expressed as mean ± SD unless otherwise indicated. In tables, different lowercase letters indicate statistically significant differences (*p* ≤ 0.05) according to the corresponding statistical test; the direction of comparison (within rows, columns, or storage time) is specified in each table footnote.

## 3. Results and Discussion

### 3.1. Characterization of Avocado Seed Flours and Wet-Milling Fractions

#### 3.1.1. Extraction Yields of Enzyme-Assisted Wet-Milling Fractions

**Figure 2:**
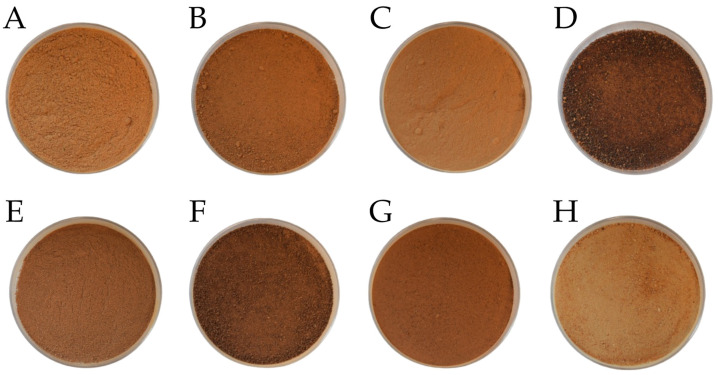
Representative images of avocado seed flours and fractions obtained after extrusion and enzyme-assisted wet milling. (**A**) Non-extruded flour (NEF); (**B**) NEF fraction 1 (NEF F1); (**C**) NEF fraction 2 (NEF F2); (**D**) NEF fraction 3 (NEF F3); (**E**) extruded flour (EF); (**F**) EF fraction 1 (EF F1); (**G**) EF fraction 2 (EF F2); and (**H**) EF fraction 3 (EF F3).

Following enzyme-assisted wet milling, three fractions were obtained: Fraction 1 (F1), corresponding to the insoluble residue retained after sieving; Fraction 2 (F2), representing the sedimented material after centrifugation; and Fraction 3 (F3), corresponding to the soluble supernatant. Representative images of the obtained flours and their corresponding fractions are presented in Figure 2. Although all fractions were characterized to understand the effect of processing on material distribution and fractionation behavior, only F1 was selected for incorporation into frankfurter-type sausages based on its techno-functional properties.

The extraction yields of enzyme-assisted wet-milling fractions obtained from non-extruded (NEF) and extruded (EF) avocado seed flours are presented in Table 2. In the NEF, Fraction 1 (NEF F1) and Fraction 2 (NEF F2) accounted for 40.24% and 44.73% of the total dry mass, respectively, whereas Fraction 3 (NEF F3) represented 15.03%. After extrusion, the distribution of mass among fractions changed. The recovery of Fraction 1 (EF F1) decreased to 30.15% (*p* ≤ 0.05), whereas Fraction 3 (EF F3) increased to 29.60%. In contrast, Fraction 2 (EF F2) yield was not significantly affected by extrusion (44.73% vs. 40.25%; *p* > 0.05). The reduction in EF F1 yield following extrusion suggests that thermomechanical processing modified the structural organization of insoluble matrix components, which may influence their separation behavior during subsequent wet milling [11,12]. Extrusion induces starch gelatinization, protein denaturation, and partial depolymerization of non-starch polysaccharides through the combined effects of heat, pressure, and shear stress [11]. These structural modifications may alter the particle size and dispersibility during wet milling through macromolecular rearrangements of the protein–fiber interactions [35]. Conversely, the significant increase in EF F3 suggests a greater proportion of soluble or fine particulate material after extrusion. This shift may be associated with fragmentation of the cell-wall polymers enhancing the solubilization of low-molecular-weight components generated after thermomechanical processing [12,36]. Similar redistribution phenomena have been described in extruded plant matrices, where processing severity alters the balance between insoluble and soluble fractions [36]. The absence of significant differences in Fraction 2 suggests that extrusion mainly affected the components associated with the most structurally resistant and the most dispersible fractions, while the intermediate fraction remains comparatively less affected. Overall, these results suggest that extrusion promotes structural reorganization of the avocado seed matrix, affecting fraction recovery during enzyme-assisted wet milling, and may influence the techno-functional properties of the resulting fractions.

#### 3.1.2. Chemical Composition of Avocado Seed Flours and Wet-Milling Fractions

The chemical composition (protein, fat, ash, and carbohydrates by difference) of NEF, EF, and the fractions obtained after enzyme-assisted wet milling (NEF F1–F3 and EF F1–F3) is summarized in Table 3. These fractions should not be considered directly comparable as equivalent treatments due to their compositional differences resulting from the fractionation process. Therefore, differences among fractions are interpreted descriptively rather than through direct statistical comparison. In the whole flour, extrusion significantly increased crude protein content (NEF: 4.51% vs. EF: 5.23%; *p* ≤ 0.05) and decreased crude fat (NEF: 1.18% vs. EF: 0.76%; *p* ≤ 0.05), while ash and carbohydrates (by difference) did not show significant changes. The increase in crude protein content can be explained by a relative compositional shift associated with the reduction in fat content, which proportionally increases the contribution of protein in the overall composition. Following extrusion, enzyme-assisted wet milling led to a redistribution of macronutrients among fractions, reflecting differences in their dispersibility and sedimentation behavior. These changes are consistent with structural modifications of plant matrices induced by thermomechanical processing, including increased porosity and alterations in hydration and solubility properties of dietary fiber components [35,37]. Additionally, extrusion may induce protein denaturation and matrix restructuring, which can influence the spatial distribution of protein within the matrix [12,35]. A reduction in apparent fat content after extrusion has been reported in several plant matrices and is often associated with thermomechanical restructuring of starch–protein–fiber networks, which may influence lipid distribution and solvent accessibility during extraction [38]. In addition, thermomechanical processing may promote lipid migration or structural entrapment within reorganized starch–protein matrices, further reducing solvent accessibility during conventional extraction procedures [38]. The dietary fiber fractions determined in the whole flours showed a consistent decrease after extrusion (TDF: 24.48% to 19.83%; IDF: 21.17% to 17.32%; SDF: 3.31% to 2.52%). These results highlight the matrix-dependence of extrusion effects on dietary fiber. For example, in extruded chokeberry pomace, total dietary fiber remained unchanged while high-molecular-weight soluble dietary fiber increased with increasing thermomechanical stress [36]. In wheat bran systems, extrusion has been shown to increase SDF and decrease IDF without affecting TDF, which has been interpreted as a redistribution between soluble and insoluble fiber fractions caused by disruption of structural polysaccharide interactions [35]. However, in the present work both SDF and IDF decreased in the whole flour. Therefore, a direct conversion of insoluble dietary fiber (IDF) into soluble dietary fiber (SDF) is not supported by the experimental pattern alone. Possible explanations include partial depolymerization of structural polysaccharides into low-molecular-weight carbohydrates that are not recovered within AOAC dietary fiber fractions, or redistribution of carbohydrate fragments into soluble components quantified within the carbohydrate-by-difference fraction. Similar structural fragmentation of polysaccharides during extrusion has been reported in plant-based matrices subjected to thermomechanical processing [35,37]. However, these mechanisms cannot be confirmed without additional structural characterization of carbohydrate polymers. After enzyme-assisted wet milling, the compositional data indicate a marked redistribution across fractions. NEF F1 and NEF F2 were characterized by high carbohydrates by difference (~92%) and low ash, whereas NEF F3 showed a marked concentration of ash, particularly in the EF-derived stream (ash: 11.97% in F3 NEF vs. 24.99% in F3 EF). This compositional redistribution reflects the separation of chemically distinct material streams, which further supports the limited direct comparability among fractions as statistically equivalent treatments. This compositional redistribution is consistent with the yield patterns described in Section 3.1.1, where extrusion increased the recovery of the most dispersible fraction (F3), suggesting that matrix disruption facilitates the release of fine particulate material and mineral-associated components during wet fractionation. Enzymatic processing can modify the chemical composition of recovered streams by selectively hydrolyzing matrix components and altering the distribution of macromolecules across fractions [39]. One possible explanation is that minerals associated with structural components of the cell wall or protein matrices become more accessible after matrix disruption and protease-mediated hydrolysis, facilitating their partitioning into the most dispersible fraction during wet milling [39]. However, this interpretation should be considered as a hypothesis, since the present study did not include specific analyses to confirm the mechanisms underlying mineral redistribution in protease-assisted wet fractionation systems. Because crude fiber provides only an approximate estimate of fibrous components, the determination of total, insoluble, and soluble dietary fiber fractions (TDF, IDF, SDF) offers a more informative assessment of process-induced changes in high-fiber matrices such as avocado seed flour [40]. The application of AOAC-based chemical composition analysis and dietary fiber quantification has also been reported in previous studies evaluating the compositional effects of extrusion processing in plant-derived flours [11,41].

#### 3.1.3. Acetogenin Content in Avocado Seed Flours

Extrusion markedly reduced the acetogenin content of avocado seed flour (Table 4). Total acetogenins decreased from 11.99 ± 1.37 mg/g (dry weight basis) in NEF to 2.07 ± 0.07 mg/g in EF, corresponding to an overall reduction of 82.77% (*p* ≤ 0.05). All quantified acetogenins showed significant reductions, with decreases ranging from 73.74% (persin) to 93.35% (AcO-avocadiene B). This consistent decline across structurally distinct molecules suggests that the reduction was not limited to specific acetogenins, but rather is consistent with a general decrease affecting multiple compounds under the applied extrusion conditions. Acetogenins have been reported to retain antimicrobial activity after static thermal treatments up to 120 °C for 15 min and under high hydrostatic pressure conditions (300–600 MPa, 3–6 min, 25 °C) [42], suggesting considerable molecular stability under non-shear processing conditions. In contrast, the magnitude of reduction observed under the present extrusion conditions exceeds those reported for static heating alone. Twin-screw extrusion combines thermal input with mechanical shear and pressure, resulting in pronounced structural reorganization of plant matrices [12,36]. Extrusion studies in plant-based materials have demonstrated that increasing specific mechanical energy (SME) and shear severity promote cell wall disruption, macromolecular restructuring, and increased accessibility of intracellular constituents [11,12,35,36]. Within this process–structure framework, the observed attenuation of acetogenins may result from enhanced molecular exposure to the combined thermal and mechanical stresses generated during high-shear extrusion. However, the available literature does not provide direct evidence identifying the specific chemical degradation pathways of acetogenins under extrusion conditions. The reduction pattern among individual acetogenins was not uniform, suggesting structure-dependent susceptibility. Persin exhibited the lowest reduction (73.74%), whereas several acetoxy-substituted derivatives, including AcO-avocadiene B and AcO-avocadenyne, showed reductions exceeding 90%. Pacheco [42] reported enhanced persistence of acetogenins bearing a 4-oxo or trans-enone functionality during storage, indicating that structural features may influence molecular stability. Persin and the persenones (A, B, C), structurally characterized by Rodríguez-Sánchez [20] and Rodríguez-López [27], contain such functionalities and exhibited comparatively lower reductions than some acetoxy-rich derivatives. Although this trend aligns with previously described structure–stability relationships, the literature does not establish a confirmed mechanistic link between specific functional groups and degradation under high-shear extrusion. Therefore, the structure-dependent attenuation observed here should be interpreted as indicative rather than mechanistically resolved. From a formulation and compositional standpoint, the attenuation of acetogenins alters the lipophilic bioactive load of the flour, a factor that may become particularly relevant in matrices where lipid partitioning and oxidative interactions contribute to product stability. Acetogenins and related oxylipins have been identified as contributors to bitter perception in avocado-derived products [43], and persin has been associated with toxicological effects in animal models at sufficiently high exposure levels [44]. Although the present study does not evaluate toxicological thresholds or exposure scenarios, extrusion achieved a marked decrease in endogenous acetogenin concentration. Importantly, these compounds were attenuated but not eliminated, indicating controlled mitigation rather than complete removal. Such modulation of bioactive load may facilitate formulation considerations when incorporating this upcycled plant ingredient into meat emulsions. Overall, the findings suggest a process–structure–chemistry interaction whereby high-shear extrusion, characterized by substantial mechanical energy input and matrix restructuring, coincides with attenuation of lipophilic endogenous bioactives. In the context of upcycled ingredients intended for animal-origin foods, extrusion may therefore function not only as a structuring technology but also as a mitigation step for naturally occurring plant bioactives. It should be noted that acetogenin content was determined only in non-extruded and extruded flours (NEF and EF), and not in the fractions obtained after enzyme-assisted wet milling. Therefore, the distribution of these compounds among F1, F2, and F3 cannot be established from the present data. Given that fractionation may influence the partitioning of lipophilic compounds, further research is required to evaluate the behavior of acetogenins in these derived fractions.

#### 3.1.4. Antioxidant Activity of Avocado Seed Flours and Fractions

Extrusion markedly reduced the antioxidant capacity of avocado seed flour. Antioxidant activity (AA) measured by the DPPH radical scavenging assay decreased from 53.34% in NEF to 19.95% in EF (*p* ≤ 0.05). These results confirm that extrusion substantially reduced the measurable antioxidant capacity of avocado seed flour. Avocado seeds are recognized as a rich source of phenolic compounds, including flavonoids and phenolic acids that contribute to their radical scavenging capacity [5,45]. Extrusion processing can modify the antioxidant profile of plant matrices, with effects depending on temperature, moisture content, and mechanical shear [7,46]. Under high thermomechanical stress, phenolic compounds may undergo transformations such as thermal degradation, polymerization, or interactions with macromolecules, which can reduce their measured radical scavenging activity [7]. Consequently, the observed reduction in antioxidant activity may reflect structural or extractability changes in antioxidant compounds induced by extrusion. Because the DPPH assay reflects the electron- or hydrogen-donating capacity of antioxidant compounds, structural modifications induced by extrusion may reduce their apparent radical scavenging activity even when antioxidant molecules remain present in the matrix [47]. Following enzyme-assisted wet milling, antioxidant activity was distributed among the obtained fractions (NEF F1–F3 and EF F1–F3), revealing differences in radical scavenging capacity relative to the whole flours. In the case of NEF, the highest DPPH inhibition was observed in the whole flour (53.34%), followed by NEF F1 (35.32%), NEF F2 (30.30%), and NEF F3 (23.90%). A generally similar decreasing pattern was observed for the EF-derived fractions, although absolute inhibition values were markedly lower than those measured in NEF. While no statistically significant differences were detected between corresponding NEF and EF fractions, likely due to variability within the data, a decreasing trend in antioxidant activity was observed in EF F2 and EF F3. Interestingly, the EF F1 exhibited a higher mean inhibition value than the corresponding extruded whole flour, suggesting that fractionation may partially concentrate antioxidant compounds in this fraction. At present, the mechanisms governing the distribution of antioxidant activity among fractions cannot be fully elucidated, since individual antioxidant compounds were not quantified in this study. Antioxidant compounds in plant matrices may occur either as free molecules or associated with structural components of the cell wall. Processing steps that disrupt cellular structures can therefore influence their extractability and measured antioxidant capacity [2,47,48,49]. In this context, the differences observed among fractions may reflect variations in the partitioning or extractability of antioxidant compounds during enzymatic wet milling, although additional compositional analyses would be required to identify the specific compounds responsible for these effects. Overall, extrusion substantially reduced the DPPH radical scavenging activity of avocado seed flour; however, measurable antioxidant capacity remained in the fractions obtained after enzymatic wet milling. Among these fractions, NEF F1 and EF F1 tended to show higher antioxidant activity compared to the other fractions, suggesting that this fraction retains a substantial proportion of radical-scavenging compounds. This finding is technologically relevant because plant-derived ingredients containing antioxidant compounds have been widely explored as functional components in meat products due to their potential contribution to oxidative stability, product quality, and nutritional value [19,50]. In this context, the retention of antioxidant activity in the NEF F1 and EF F1 supports its potential use as an upcycled functional ingredient in meat emulsions. Nevertheless, further studies evaluating the behaviour of these compounds within the meat matrix would be necessary to fully elucidate their functional impact.

#### 3.1.5. Water Absorption and Oil Absorption Indices

WAI and OAI were determined to evaluate the hydration and lipid-binding behavior of avocado seed flour (NEF and EF) and the fractions obtained after enzyme-assisted wet milling (NEF F1–F3 and EF F1–F3) (Table 5). Extrusion significantly increased the WAI of the whole flour and all recovered fractions (*p* ≤ 0.05). In the whole flour, WAI increased from 2.87 to 3.91 g/g (NEF vs. EF). A similar trend was observed across all fractions, with values increasing from 3.57 to 3.99 g/g in F1, from 2.55 to 3.79 g/g in F2, and from 0.50 to 1.27 g/g in F3. In contrast, OAI exhibited the opposite trend, decreasing after extrusion in the whole flour from 2.12 to 1.84 g/g (*p* ≤ 0.05). Likewise, OAI decreased in F1 (2.31 to 1.96 g/g) and F2 (2.16 to 1.81 g/g), whereas no significant change was observed in F3 (1.95 vs. 1.82 g/g; *p* > 0.05). Similar trends involving enhanced hydration properties after thermomechanical processing have been reported in plant-derived fiber systems and have been associated with structural reorganization of the biopolymer matrix during extrusion [35,51]. Such processing can generate more porous structures and expose hydrophilic functional groups, thereby increasing water-binding capacity and modifying interactions with lipids [35,51]. The consistent increase in WAI across the whole flour and all fractions indicates that thermomechanical processing modified the hydration behavior of the avocado seed matrix. Similar effects have been widely reported in extruded plant-based materials and are generally attributed to structural transformations induced by heat, pressure, and shear, which generate more porous structures and expose hydrophilic functional groups capable of interacting with water [35,51]. In contrast, the reduction in OAI following extrusion indicates a concurrent modification of lipid-binding interactions within the matrix. Comparable trends have been reported in extruded cereal–legume systems, where extrusion increased water interaction properties while reducing oil-holding capacity [35]. These responses have been associated with structural rearrangements of biopolymers and changes in the balance between hydrophilic and hydrophobic interactions within the matrix, which can modify surface characteristics and lipid retention behavior during functional testing. Beyond the extrusion effect, differences in WAI and OAI among the recovered fractions further indicate that enzyme-assisted wet milling redistributed components of the avocado seed matrix into streams with distinct techno-functional behaviors. Fractions F1 and F2, characterized by high carbohydrate contents (~92%) and relatively low ash levels, tended to show higher WAI values compared to F3, suggesting that these carbohydrate-rich fractions may contribute more strongly to hydration-related functionality. In contrast, F3 showed comparatively lower WAI values in both NEF and EF materials, indicating limited interaction with water relative to the other fractions. Hydration-related functional properties of plant-derived ingredients are strongly influenced by structural attributes of dietary fiber and carbohydrate-rich matrices, including porosity, particle morphology, and solubility, which determine the availability of hydrophilic sites for water binding [51]. In addition, structural modifications generated during enzymatic processing or fiber disintegration can alter hydration and oil-retention properties by exposing additional binding sites and modifying the organization of cell-wall polysaccharides [39]. Recent studies have also reported that enzymatic modification of plant dietary fibers can increase water- and oil-related functional properties in parallel with the development of more porous structures and reduced crystallinity [52], while combined thermal and enzymatic treatments have been shown to modify both water- and oil-retention capacities in plant fiber matrices [14]. These considerations are consistent with the functional differences observed among the avocado seed fractions. Among the recovered streams, F1 combined the highest WAI with relatively elevated OAI values compared with the other fractions, suggesting balanced hydration and lipid-binding behavior. Such combined functional properties are generally considered desirable attributes in plant-derived ingredients intended for use in complex food matrices, including emulsified systems. Plant-based ingredients incorporated into frankfurter formulations contribute to product functionality through their ability to interact with both water and fat phases. Dietary fibers and carbohydrate-rich plant matrices have been reported to improve water retention and contribute to structural stabilization in processed meat products through interactions with the aqueous phase and dispersed fat droplets [18,19,47]. In addition, recent reviews on dietary fibers and plant-based ingredients in meat and meat-analogue systems emphasize that hydration and oil-binding properties play a key role in determining the structural behavior of these ingredients in complex food matrices [53]. In this context, the relatively high WAI values observed for F1, together with its moderate oil absorption capacity, suggest that this fraction may provide a favorable balance of hydration and lipid-binding interactions when incorporated into meat emulsions. Although the present results represent ingredient-level functionality measured under standardized conditions, these properties provide an indication of the potential technological behavior of the avocado seed fractions in complex food systems. Their actual technological impact was subsequently evaluated in frankfurter-type sausages formulated with these ingredients.

#### 3.1.6. Emulsifying Capacity and Emulsion Stability of Avocado Seed Flours

The EC and ES of avocado seed flours and their fractions are presented in Table 5. Overall, EC values ranged from 41.88 to 45.45%, indicating similar emulsifying performance across the samples evaluated. At the whole-flour level, extrusion had no significant effect on EC, with values of 43.86% and 42.97% for NEF and EF, respectively. A similar behavior was observed for most fractions obtained after enzyme-assisted wet milling, suggesting that the combination of extrusion and wet fractionation did not substantially alter the emulsifying capacity of the flour matrix. The only statistically significant difference was observed in Fraction F1, where EC increased from 42.45% in NEF F1 to 44.81% in EF F1. This result is particularly relevant from a technological perspective, as fraction F1 was the ingredient selected for incorporation into frankfurter-type sausages. The relatively small variation in EC among samples may be partially explained by the compositional characteristics of the fractions, which were predominantly composed of carbohydrates (≈67–92%) and contained relatively low protein levels (≈3–6%). Previous studies have reported that low protein content may restrict the emulsifying capacity of plant-derived fiber matrices, as proteins typically represent the main surface-active components responsible for stabilizing oil–water interfaces [39]. Although extrusion significantly increased WAI (Table 5), this change was not accompanied by substantial modifications in emulsifying capacity. Similar behavior has been reported in modified dietary fiber systems, where improvements in hydration-related properties do not necessarily translate into enhanced interfacial activity [39]. Structural changes induced by extrusion, including disruption of macromolecular organization, reduction in particle size, and the formation of more porous matrices, have been documented in plant-derived fiber materials and are generally associated with improved hydration properties rather than direct enhancement of interfacial activity [14,35,37]. The slight increase in EC observed for F1 after extrusion suggests that structural modifications generated during thermomechanical processing may become functionally relevant after the wet-milling fractionation step. The enzyme-assisted treatment followed by sieving and centrifugation likely promoted the separation of matrix components with different physicochemical characteristics, potentially influencing the ability of the retained fraction to participate in emulsion formation. However, there is insufficient evidence in the provided literature to attribute this behavior to a specific mechanistic pathway.

In contrast to EC, emulsion stability values ranged from approximately 40.8 to 43.6% and did not differ significantly between NEF and EF for any of the evaluated fractions. These results indicate that neither extrusion nor enzyme-assisted wet milling compromised the ability of avocado seed flour to maintain emulsion stability after thermal treatment. The preservation of ES despite the structural modifications induced by processing suggests that the processing treatments did not substantially affect the ability of the matrix to stabilize emulsions under the evaluated conditions. This observation is technologically relevant, as stable oil–water emulsions are essential for the structural stability and quality attributes of emulsified meat products such as sausages. In these systems, the stabilization of dispersed fat droplets strongly influences the final texture and product integrity. Previous studies have shown that plant-derived ingredients, including plant proteins and dietary fibers, can contribute to the stabilization of emulsified meat systems through their ability to interact with water and lipid phases and to participate in the formation of structural networks within the matrix [51,54]. Therefore, the preservation of emulsifying functionality observed for the avocado seed fractions, particularly F1, suggests that the ingredient may retain its technological functionality when incorporated into complex food matrices such as frankfurter-type meat emulsions.

#### 3.1.7. Pasting Properties of Avocado Seed Flours (RVA)

**Figure 3:**
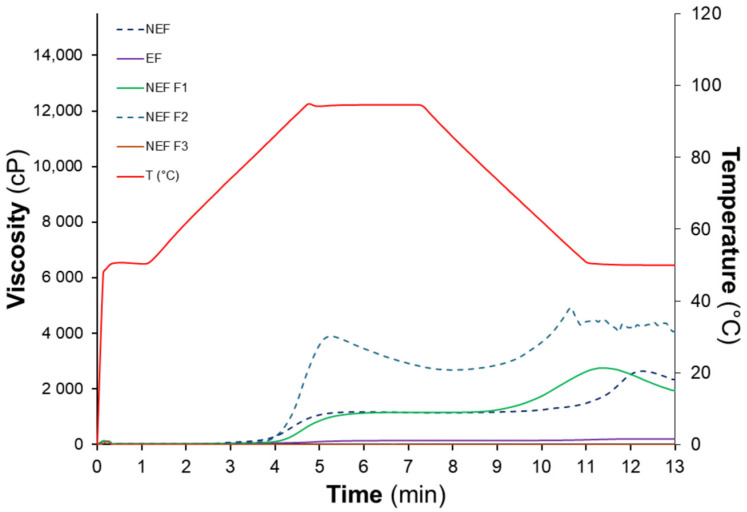
Rapid Visco Analyzer (RVA) pasting profiles of avocado seed flours and selected wet-milling fractions as a function of temperature during a standard heating–cooling cycle. Samples include non-extruded flour (NEF), extruded flour (EF), and enzyme-assisted wet-milled fractions (NEF F1, NEF F2, and NEF F3).

The pasting profiles of avocado seed flours and their fractions revealed pronounced differences (Figure 3), indicating that enzyme-assisted wet milling and extrusion substantially modified viscosity development during the heating–cooling cycle. The quantitative pasting parameters obtained from the curves are summarized in Table 6. Among the non-extruded materials, the NEF F2 fraction exhibited markedly higher peak viscosity (PV), trough viscosity (TV), final viscosity (FV), and setback (SB) compared with both the whole flour (NEF) and fraction NEF F1. In contrast, the extruded flour (EF) displayed a markedly reduced viscosity profile, characterized by very low PV, TV, FV, and SB values and the absence of a detectable pasting temperature. Within the non-extruded materials, NEF2 showed the highest viscosity development (*p* ≤ 0.05) during both heating and cooling stages, with PV and FV values of 3881 and 4509 cP, respectively, which were approximately three times higher than those of the whole flour (NEF), as illustrated by the pronounced viscosity peak in Figure 3. The breakdown value of NEF2 (1210 cP) was also substantially greater than those of NEF and NEF F1, indicating greater susceptibility of swollen starch granules to shear-induced disintegration. This behavior suggests that enzyme-assisted wet milling generated a fraction containing components that strongly contribute to viscosity development. The pasting properties of plant flours are known to depend strongly on the relative proportion of starch and non-starch constituents, as components such as dietary fiber may restrict starch swelling and limit viscosity development during heating [6]. Although starch content was not directly quantified in each fraction, the markedly higher viscosity parameters observed for NEF F2 suggest that fractionation concentrated components contributing to viscosity generation during gelatinization. In contrast, NEF1 exhibited PV and TV values comparable to those of NEF but showed lower breakdown and setback values, together with a longer peak time and higher pasting temperature. These characteristics suggest slower viscosity development and greater resistance to viscosity loss during the high-temperature holding stage. Interactions between starch and other matrix components, including dietary fiber or protein, have been reported to limit granule swelling and modify pasting behavior in composite plant flours [6,55]. However, the available literature does not provide sufficient evidence to attribute the specific RVA behavior of NEF F1 to a particular compositional mechanism, and further compositional characterization would be required to clarify the structural basis of this response. Extrusion had the most pronounced impact on pasting behavior. The extruded flour (EF) exhibited extremely low viscosity throughout the RVA cycle (PV 138 cP; FV 193 cP), together with minimal breakdown and setback values and the absence of a measurable pasting temperature. Similar reductions in viscosity parameters have been widely reported in extruded starch-rich systems and are generally attributed to thermomechanical disruption of starch granules, including partial gelatinization and depolymerization, which reduce their ability to swell and develop viscosity during subsequent heating [55,56]. In agreement with these mechanisms, the near suppression of viscosity in EF suggests that the structural integrity of the starch fraction in avocado seed flour was extensively altered during extrusion. Overall, the results demonstrate that the processing sequence applied in this study markedly modified the pasting behavior of avocado seed flour. Enzyme-assisted wet milling produced fractions with highly contrasting viscosity profiles, likely reflecting redistribution of matrix components, whereas extrusion drastically reduced viscosity development, suggesting extensive structural modification of starch. These structural and rheological differences may partially explain the variations observed in water absorption and emulsion stability reported in the previous sections. From a technological perspective, ingredients capable of developing higher viscosity during heating, such as NEF F2, may contribute to water immobilization and structural reinforcement in complex food matrices, whereas materials with severely reduced viscosity development, such as EF, may behave primarily as low-viscosity structural components. Consequently, the marked differences in RVA profiles among the avocado seed fractions may influence their technological functionality when incorporated into frankfurter-type emulsified meat systems.

#### 3.1.8. In Vitro Protein Digestibility of Avocado Seed Flours

In vitro protein digestibility (IVPD) of NEF and EF avocado seed flours and their enzyme-assisted wet-milling fractions is presented in Table 7. IVPD values ranged from 70.25% to 76.64% across samples, and the effect of extrusion varied among fractions. For the whole flour, extrusion reduced IVPD from 76.64% in NEF to 72.90% in EF (*p* ≤ 0.05). A similar decrease was observed in NEF F2 and EF F2 (75.49% vs. 71.21%). In contrast, the NEF F1 exhibited higher digestibility, increasing from 72.78% in NEF to 75.19% in EF, whereas no significant differences between treatments were detected for F3. Overall, F3 exhibited the lowest digestibility values (~70%), while the highest IVPD values were observed in NEF whole flour and EF F1. A similar pattern was observed when digestibility was expressed relative to casein. Relative protein digestibility ranged from 25.56 to 58.15%, with whole NEF presenting the highest value and F3 the lowest in both treatments. Extrusion reduced relative digestibility in the whole flour (58.15% to 41.53%; *p* ≤ 0.05) and in F2 (53.36% to 30.99%; *p* ≤ 0.05), while markedly increasing digestibility in F1 (38.98% to 51.76%). No significant differences between NEF F3 and EF F3 were detected. These results indicate that the impact of extrusion on protein digestibility was not uniform across the system and depended on the compositional and structural characteristics of the fractions generated during enzyme-assisted wet milling. The distinct digestibility patterns observed among fractions are consistent with the compositional differences reported in Table 2. F1 and F2 were characterized by a predominance of carbohydrate-derived components, whereas F3 exhibited the lowest protein content and a comparatively higher mineral fraction. Such compositional heterogeneity suggests that proteins may be embedded within plant matrices of different structural organization, which can influence their susceptibility to enzymatic hydrolysis. The increase in digestibility observed for EF F1 suggests that extrusion may have altered the structural organization of proteins within this fraction, potentially facilitating enzymatic access during digestion. Thermomechanical processing can influence protein digestibility by modifying protein conformation and matrix structure, potentially enhancing enzyme accessibility [11,56]. In addition, thermomechanical treatments have been reported to modify plant cell wall polysaccharides and disrupt the microstructure of fiber-rich matrices, generating more porous matrices and altering the organization of surrounding biopolymers [35,36]. These structural changes may indirectly influence protein accessibility within plant-based matrices. However, the direction and magnitude of these effects depend strongly on matrix composition. In plant-derived systems rich in phenolic compounds, interactions between proteins and polyphenols may reduce proteolytic susceptibility and limit digestibility [57,58,59]. Additionally, enzyme-assisted wet milling can generate fractions with distinct particle size distributions and microstructural organization that influence enzymatic accessibility during digestion [32]. Therefore, the higher digestibility observed in EF-F1 may reflect structural modifications induced by extrusion that become more evident after fractionation, whereas the lower digestibility observed in the whole flour and NEF F2/EF F2 suggests that matrix interactions may limit enzymatic hydrolysis in these materials. Nevertheless, because protein structural changes and protein–matrix interactions were not directly evaluated in this study, the specific mechanisms underlying these differences cannot be conclusively established.

### 3.2. Frankfurter Formulation and Technological Quality During Refrigerated Storage

#### 3.2.1. Chemical Composition of Frankfurter Samples

The chemical composition and energy value of CNF, SNF, and SEF frankfurters at 0 and 35 days of refrigerated storage (4 ± 1 °C) are shown in Table 8. Two-way ANOVA revealed that several compositional parameters were not significantly affected by treatment, storage time, or their interaction. Moisture, protein, total dietary fiber (TDF), insoluble dietary fiber (IDF), soluble dietary fiber (SDF), and energy value showed no significant effects (*p* > 0.05) for treatment (T), storage time (S), or the T × S interaction. In contrast, fat content was significantly affected by both treatment and storage time (*p* < 0.001), whereas ash and carbohydrate contents were influenced by treatment (*p* < 0.05) and storage (*p* < 0.001 and *p* < 0.01, respectively). The absence of significant T × S interactions indicates that storage-related changes followed similar trends across all formulations. The absence of treatment effects on moisture and protein indicates that replacing soy fiber with avocado seed flour ingredients did not significantly modify the main compositional fractions of the frankfurters. This outcome is consistent with the formulation strategy, as the dietary fiber ingredient represented only 1% (*w*/*w*) of the total batter, while the base formulation, including turkey meat, water, starches, and soy protein concentrate, remained identical across treatments. In emulsified meat systems, plant-derived fiber ingredients may influence water retention due to their capacity to bind water and interact with the protein matrix, contributing to the stabilization of the emulsion structure [60]. In this context, the relatively high-water absorption index (WAI) previously observed for the avocado seed flour fractions may contribute to water retention in the batter system. Similarly, the dietary fiber fractions measured in the finished products (TDF, IDF, and SDF) did not differ significantly among treatments or storage times. However, because the dietary fiber composition of the F1 fraction incorporated into the sausages was not directly determined, it is not possible to establish whether the added ingredient contributed measurable changes to fiber fractions in the final product. Significant treatment effects were observed for fat, ash, and carbohydrate contents. At both storage times, the CNF exhibited slightly higher fat content than SNF and SEF, whereas the latter two treatments did not differ significantly from each other. These differences were minor and likely reflect minor compositional contributions of the dietary fiber ingredients, considering their low inclusion level (1% *w*/*w*) in the sausage formulation. In reformulated meat products, changes in the chemical composition are often associated with the intrinsic chemical composition of the incorporated ingredient, particularly when plant-derived materials rich in carbohydrates and minerals are used [17,61]. In the present study, the high carbohydrate proportion reported for the F1 fractions of avocado seed flour may partially contribute to the higher carbohydrate values observed in SNF and SEF compared with the control formulation. It should also be noted that carbohydrates were calculated by difference; therefore, variations in other components (moisture, protein, fat, ash, and fiber) may influence the calculated carbohydrate values. From a functional standpoint, the oil absorption capacity and emulsifying properties of dietary fiber ingredients may influence lipid distribution within the meat emulsion matrices, as these properties are associated with the stabilization of oil–water systems in processed meat products [14,47,61]. However, the available evidence does not allow a direct causal attribution of the observed fat differences to these mechanisms. Storage time significantly affected fat, ash, and carbohydrate contents, although the magnitude of these changes was relatively small. Fat and ash contents decreased slightly after 35 days of refrigerated storage, whereas carbohydrate values increased across all formulations. Because the interaction between treatment and storage time was not significant for any variable, these storage-related changes occurred similarly in all treatments. Overall, these results indicate that the incorporation of avocado seed flour, either NEF or EF, did not compromise the compositional stability of the frankfurters during refrigerated storage. The substitution of soy fiber with avocado seed flour maintained the general compositional profile and energy value of the product while introducing only minor variations in specific proximate fractions, supporting the technological feasibility of incorporating this upcycled plant ingredient into emulsified meat products [18,62].

#### 3.2.2. Instrumental Color

Instrumental color parameters (L*, a*, b*, chroma, and hue angle) were significantly affected by treatment (T), storage time (S), and their interaction (T × S) (*p* ≤ 0.05) (Table 9). The significance of the interaction term indicates that the evolution of instrumental color during refrigerated storage depended on the formulation, meaning that the three frankfurter systems followed distinct chromatic trajectories over time. Lightness (L*) showed consistent differences among formulations throughout storage. The control formulation (CNF) exhibited higher L* values than sausages containing avocado seed flour (SNF and SEF) at all evaluated times. At day 0, CNF presented an L* value of 66.48, whereas SNF and SEF showed lower values (57.91 and 57.60, respectively), indicating that replacing soy fiber with avocado seed flour produced darker frankfurters from the beginning of storage. Similar reductions in lightness have been reported in reformulated frankfurters containing plant-derived ingredients, reflecting the optical contribution of these materials to the meat matrix [17,41,63]. Likewise, the incorporation of plant-derived ingredients or plant proteins, including extruded materials, has been reported to significantly influence L* values in hybrid or plant-enriched sausages [54,63]. The redness parameter (a*) showed a more variable response during storage. At several storage points, particularly days 0, 14, and 28, sausages formulated with avocado seed flour exhibited higher a* values than the control, although these differences were not maintained across the entire storage period. This temporal variability reflects the formulation-dependent color evolution observed during storage. In cured meat products, the a* coordinate is commonly associated with the contribution of red meat pigments and their transformations during storage, including reactions involving cured pigments and oxidative processes affecting myoglobin derivatives and lipid components [17]. Changes in redness have also been reported in emulsified sausages formulated with plant-derived ingredients or natural pigments, which may influence the intensity of red color through interactions with meat pigments and oxidative processes within the matrix [17,63]. The observed differences in redness (a) among formulations during storage may reflect formulation-dependent oxidative processes within the meat matrix. In cured meat systems, changes in redness are associated with transformations of myoglobin derivatives and cured pigments, which can be influenced by lipid oxidation and interactions with added ingredients [18]. In the control formulation (CNF), the increase in redness observed at specific storage times may be related to pigment stabilization or redistribution processes occurring during storage. In contrast, the different behavior observed in SNF and SEF formulations may be associated with the presence of plant-derived compounds, including phenolic constituents, which could influence oxidative reactions within the matrix [17]. However, the present study does not include direct measurements of lipid oxidation or pigment stability, and therefore these interpretations should be considered indicative rather than mechanistically confirmed. However, the present data do not allow attribution of the observed variations to a specific pigment-related mechanism. Clearer formulation-related differences were observed for yellowness (b*). At the beginning of storage (days 0 and 14), the treatments followed the order SNF > SEF > CNF, indicating that the incorporation of avocado seed flour increased yellowness relative to the control. Although this pattern changed at intermediate storage times, SNF generally maintained higher b* values than CNF throughout storage. Similar increases in b* have been reported in reformulated frankfurters containing plant coproducts or plant-derived ingredients, reflecting the optical contribution of the added materials [17,54,63]. Comparable increases in yellowness have also been reported in frankfurter-type sausages formulated with plant-derived ingredients such as chia coproducts, where pigments present in the plant material increased the b* coordinate [17]. Chroma (C*) followed the trends observed for a* and b*, reflecting overall color saturation derived from these coordinates. Sausages containing avocado seed flour generally exhibited higher C* values than the control at several storage times, particularly at days 0, 14, and 28, indicating greater color intensity in the reformulated products. According to Delgado-Ospina [18], chroma reflects the visual saturation of color in meat products because it is calculated from the combination of a* and b* coordinates. Hue angle (h°) showed comparatively smaller differences among treatments. Although some significant differences were detected at specific storage times, overall variations were moderate. Because hue angle is mathematically derived from the ratio of b* to a* values, its behavior reflects the relative balance between these coordinates [18], which changed during storage in a formulation-dependent manner. The significant treatment × storage interaction indicates that changes over time were formulation-dependent and are therefore interpreted based on the combined effect of these factors rather than on isolated comparisons within each treatment. Overall, replacing soy fiber with avocado seed flour modified the instrumental color of frankfurter-type sausages both at the initial stage and during refrigerated storage. The significant treatment × storage interaction indicates that each formulation followed a distinct chromatic evolution over time, suggesting that the incorporation of avocado seed flour and storage conditions jointly influenced the optical properties of the products. Similar formulation-dependent changes in the physicochemical and color characteristics of frankfurter-type sausages have been reported when plant-based ingredients or agro-industrial byproducts are incorporated into the meat matrix [18]. It should be noted that color measurements were performed on the final product, and not on the individual ingredients. Therefore, the observed differences in color parameters reflect the combined effects of ingredient incorporation, meat matrix interactions, and processing conditions, rather than the intrinsic color of the added materials alone.

#### 3.2.3. pH and Water Activity of Frankfurter Samples

The pH and water activity (Aw) are key physicochemical parameters influencing the stability of emulsified meat products during storage. Their evolution in frankfurter-type sausages formulated with avocado seed flour is presented in Table 10. Two-way ANOVA revealed significant effects of treatment (T), storage time (S), and their interaction (T × S) on pH (*p* < 0.001), whereas Aw was not affected by these factors (*p* > 0.05). At day 0, SNF exhibited the highest pH (6.52), followed by CNF (6.26), whereas SEF showed the lowest value (6.13). During refrigerated storage, pH changes differed among formulations, with SNF showing a decreasing trend, whereas CNF and SEF exhibited slight increases over time, reflecting a formulation-dependent evolution of this parameter. By days 28 and 35, the pH values of CNF and SNF were no longer significantly different, while SEF remained consistently lower than the other treatments. The significant treatment × storage interaction indicates that pH evolution during storage was dependent on formulation and is therefore interpreted based on the combined effect of these factors rather than on isolated comparisons within each treatment. Changes in pH during refrigerated storage have been reported in frankfurter-type sausages and other emulsified meat products reformulated with plant-derived ingredients. For example, Fernández-López [17] reported gradual pH decreases in frankfurters formulated with fruit byproducts during storage. Similar trends have also been described in reformulated frankfurter systems containing plant fibers and agro-industrial byproducts, where the incorporation of plant matrices can influence physicochemical changes occurring during storage [18,19]. In the present study, the differences observed between SNF and SEF may reflect compositional and structural differences between the non-extruded and extruded avocado seed flours used in the formulations. As discussed in Section 3.1, extrusion modified the physicochemical characteristics of the flour, particularly its hydration behavior and structural organization, which may influence interactions within the meat emulsion during storage. However, the present results do not allow identification of a specific mechanism explaining the observed pH differences.

In contrast, water activity remained stable across treatments and storage times, with values close to unity throughout the 35-day storage period. Similar stability has been reported in emulsified meat products reformulated with dietary fibers, where fiber incorporation does not necessarily modify equilibrium moisture conditions [18,64]. The absence of significant differences suggests that replacing soy fiber with avocado seed flour, either in native or extruded form, did not alter the overall availability of water within the sausage matrix. Overall, the combined results for pH and Aw indicate that although formulation influenced the evolution of pH during storage, the incorporation of avocado seed flour did not compromise the physicochemical stability of the frankfurter matrix under refrigerated conditions. It should be noted that the pH of the individual ingredients (soy fiber, NEF, and EF) was not determined in the present study. Therefore, the observed differences in pH among formulations cannot be directly attributed to the intrinsic pH of the added materials, but rather reflect the combined effects of ingredient incorporation, matrix interactions, and storage-related changes.

#### 3.2.4. Cooking Loss of Frankfurter Samples

Cooking loss is a key technological parameter used to evaluate water and fat retention in emulsified meat products, reflecting the ability of the protein–fat–water matrix to retain fluids during thermal processing. The evolution of cooking loss in frankfurter-type sausages formulated with avocado seed flour during refrigerated storage is presented in Table 10. Overall, cooking loss ranged from 1.67 to 3.77%, indicating relatively low processing losses across all treatments compared with values typically reported for emulsified sausages [41,60]. At day 0, CNF exhibited the highest cooking loss (3.77%), whereas SNF showed the lowest value (2.44%) and SEF presented intermediate values (3.20%). During the early storage period (7–14 days), both SNF and SEF exhibited significantly lower cooking loss than CNF (*p* ≤ 0.05). At day 21, SNF remained significantly lower than CNF, whereas SEF showed intermediate values without statistical differences from the other treatments. In contrast, no significant differences among formulations were detected at 28 and 35 days, when cooking loss values converged to approximately 1.7–2.3%. Two-way ANOVA revealed significant effects of treatment (T), storage time (S), and their interaction (T × S) (*p* < 0.001). The significant treatment × storage interaction indicates that the evolution of cooking loss during storage was formulation-dependent and should be interpreted considering the combined effect of these factors rather than isolated within-treatment comparisons. Because the interaction was significant, cooking loss is interpreted based on the combined influence of treatment and storage time, with comparisons among treatments at each storage time used to support the formulation-dependent behavior of the system. Differences among formulations were mainly observed at early storage stages (0–21 days), whereas cooking loss values converged at later stages. The technological behavior observed among formulations may be interpreted considering the functional properties of the avocado seed ingredients. As discussed in Section 3.1, extrusion and enzyme-assisted wet milling modified the functional properties of avocado seed flour, particularly its hydration and lipid-binding behavior. In the present study, the lower cooking losses observed for SNF during the early stages of storage may reflect the capacity of fiber- and carbohydrate-rich plant matrices to interact with the aqueous phase and contribute to moisture retention within the meat matrix. Similar reductions in cooking loss have been reported in frankfurter-type sausages formulated with plant-derived ingredients rich in dietary fiber, where treatments containing vegetable matrices exhibited lower processing losses than control formulations [41,60]. Although extrusion increased the WAI of avocado seed flour (Table 5), the SEF formulation did not consistently exhibit lower cooking loss than SNF. This suggests that technological performance in emulsified systems depends on multiple functional properties rather than hydration capacity alone. The extrusion simultaneously reduced the oil absorption index (OAI), indicating modifications in the balance between water-binding and lipid-retention interactions within the matrix. Previous studies have shown that the performance of plant-based ingredients in meat emulsions is influenced by their combined interactions with the aqueous phase, fat droplets, and the surrounding protein network [18,65]. Consequently, the technological effects of plant-derived ingredients cannot be inferred solely from hydration properties, as structural modifications may influence water and fat retention in different ways. It is also important to consider that avocado seed ingredients were incorporated at a relatively low substitution level (1%, *w*/*w*), replacing soy fiber in the control formulation. At such levels, the structural stability of the emulsion is primarily governed by the myofibrillar protein network and the overall formulation composition. In this context, the relatively small differences observed among treatments and the convergence of cooking loss values during the later stages of storage likely reflect the dominant contribution of the meat protein network to processing yield. Similar observations have been reported in emulsified meat products containing plant byproducts, where moderate inclusion levels resulted in technological performance comparable to conventional formulations [18,54,66]. Overall, the results demonstrate that the incorporation of avocado seed-derived ingredients obtained through extrusion and enzyme-assisted wet milling did not compromise cooking yield in frankfurter-type sausages. Cooking loss remained low and comparable to that of the control formulation containing commercial soy fiber, supporting the technological feasibility of using these upcycled ingredients as functional components in emulsified meat systems. Because water and fat retention during thermal processing contribute directly to the structural integrity of the protein–fat gel matrix, these results may also influence the mechanical properties of the final product. Therefore, instrumental hardness was further evaluated to determine whether the observed differences in cooking loss were associated with changes in the textural behavior of the frankfurter formulations during storage.

#### 3.2.5. Instrumental Hardness

Instrumental hardness of frankfurter-type sausages was significantly affected by treatment (T), storage time (S), and their interaction (T × S) (*p* < 0.001), indicating formulation-dependent changes in mechanical resistance during refrigerated storage (Table 10). The significant treatment × storage interaction indicates that hardness evolution was formulation-dependent and is therefore interpreted based on the combined effect of these factors rather than on isolated within-treatment comparisons. At day 0, no significant differences were observed among CNF, SNF, and SEF, with hardness values ranging from 1.17 to 1.23 N. However, the evolution of hardness during storage differed among formulations. The control formulation (CNF) showed a gradual decrease in hardness over time, reaching the lowest value at day 28 (1.01 N). In contrast, sausages formulated with avocado seed flour (SNF and SEF) exhibited higher hardness values from day 7 onward and reached maximum values at day 21 (1.40 and 1.41 N, respectively). Although a slight decrease occurred thereafter, both formulations maintained higher hardness values than the control at the end of the evaluated storage period (day 28). These formulation-dependent trends in hardness during storage may be associated with structural changes occurring within the protein–fat–water matrix. The gradual decrease observed in CNF may reflect progressive weakening of the protein network and redistribution of water within the matrix during storage, phenomena that have been reported in emulsified meat systems [18,61]. In contrast, the higher hardness values observed in SNF and SEF may be related to the presence of fiber- and carbohydrate-rich plant components, which can interact with the meat protein network and enhance water retention, thereby contributing to increased mechanical resistance of the matrix [18,41]. However, given that structural changes were not directly measured, these interpretations should be considered indicative rather than mechanistically confirmed. As previously discussed, processing modified the functional properties of avocado seed flour, including hydration behavior and interactions with surrounding biopolymers, which may influence the structure of emulsified meat systems. The changes in hardness of emulsified meat products containing plant-derived ingredients have been widely reported, often showing increased compression resistance due to interactions between fibrous components and the meat protein network [18,61]. Similarly, Araujo-Chapa [41] reported that the addition of fiber-rich ingredients modified the mechanical resistance of frankfurters during storage, reflecting structural changes within the product matrix. However, the textural response of emulsified meat systems to plant-based ingredients is not uniform. Fernández-López [17] showed that different chia-based ingredients produced contrasting effects on frankfurter hardness depending on their physical characteristics and functional behavior within the product structure. Other studies have also suggested that plant-derived components may alter protein–polysaccharide interactions within the emulsion matrix, affecting compression resistance and structural organization [67]. Conversely, partial substitution of meat proteins with plant ingredients in hybrid sausages has sometimes been associated with softer textures due to weakening of the protein network [63,64,68]. Therefore, the differences observed among treatments likely reflect localized structural interactions between the avocado seed ingredients and the meat system rather than large-scale modifications of the emulsion structure. Overall, the results indicate that the incorporation of avocado seed flour, in both native and extruded forms, contributed to maintaining or increasing the mechanical resistance of frankfurter sausages during refrigerated storage compared with the control formulated with commercial soy fiber. These findings suggest that avocado seed ingredients interacted differently with the sausage matrix, resulting in a distinct evolution of hardness during storage. Nevertheless, considering the variability reported in the literature, the mechanisms underlying these changes should be interpreted cautiously, as the textural behavior of reformulated emulsified meat products depends on multiple factors related to ingredient functionality and formulation composition.

#### 3.2.6. Consumer Sensory Evaluation of Frankfurter Samples

Consumer acceptance was evaluated to determine whether the incorporation of avocado seed flour influenced product perception. Consumer liking of frankfurters formulated with commercial soy fiber (CNF), non-extruded avocado seed flour (SNF), and extruded avocado seed flour (SEF) was evaluated by 106 untrained consumers using a 9-point hedonic scale (Table 11). Significant differences among formulations were observed for flavor and overall acceptability, whereas texture and freshness did not differ among samples. SNF and SEF showed higher flavor scores [5.00 (5.00–8.00)] than CNF [4.00 (4.00–7.00)], while identical median values were observed for texture [7.00 (5.00–8.00)] and freshness [7.00 (6.00–8.00)] across formulations. For overall acceptability, SEF obtained the highest median score [7.00 (6.00–8.00)], significantly exceeding CNF [6.00 (5.00–7.00)], whereas SNF showed intermediate values that did not differ significantly from either CNF or SEF. The absence of significant differences in perceived texture indicates that the incorporation of avocado seed flour did not adversely affect the structural attributes of the frankfurter matrix from a consumer perspective [63]. The higher flavor scores obtained for SNF and SEF indicate that the inclusion of avocado seed flour did not compromise flavor acceptance and may contribute positively to consumer perception relative to the control formulation. In contrast, reductions in sensory scores have been reported in some frankfurter formulations containing plant-derived ingredients, often associated with undesirable flavor or texture modifications [69]. In the present study, however, the median scores for all attributes remained above the neutral point of the hedonic scale (score = 5), indicating generally positive consumer acceptance of the reformulated products. These results are consistent with previous studies showing that moderate incorporation of plant-based ingredients can maintain sensory quality in processed meat products when formulations are properly optimized [19,68,70]. It should be noted that the consumer panel in this study was composed primarily of young adults, which may limit the generalizability of the sensory results to broader populations. Consumer preferences for meat products can vary depending on age, dietary habits, and cultural background. Therefore, the acceptability results reported here should be interpreted within the context of the evaluated demographic group. Overall, the results demonstrate that avocado seed flour, particularly after extrusion processing, can be incorporated into frankfurter-type sausages without compromising consumer acceptance, supporting its potential as a value-added ingredient for the reformulation of emulsified meat products.

## 4. Conclusions

This study demonstrates the feasibility of valorizing avocado seed, an abundant agro-industrial by-product, as a functional ingredient for emulsified meat products through a combined strategy of extrusion and enzyme-assisted wet milling. Extrusion significantly modified the structural and functional properties of avocado seed flour, increasing hydration capacity, reducing oil absorption, and markedly decreasing acetogenin content. These process-induced modifications, together with enzyme-assisted wet-milling fractionation, generated ingredient streams with differentiated techno-functional properties suitable for incorporation into complex food matrices. When incorporated at 1% (*w*/*w*) as a replacement for commercial soy fiber in frankfurter-type sausages, avocado seed flour did not compromise product quality. The reformulated sausages exhibited comparable physicochemical stability, low cooking losses, and stable textural resistance during 35 days of refrigerated storage. Consumer evaluation further indicated that the inclusion of avocado seed flour did not negatively affect product perception within the evaluated consumer group, with formulations containing avocado seed flour showing flavor and overall liking scores comparable to those of the control under the conditions of this study. From a technological perspective, the use of extrusion as a preconditioning step offers a scalable and industry-compatible approach for the transformation of fruit-processing residues into functional food ingredients. The results therefore highlight the potential of extruded avocado seed flour as an upcycled ingredient for the reformulation of emulsified meat products within a circular bioeconomy framework. However, this contribution is based on the valorization of an underutilized agro-industrial byproduct, and no quantitative assessment of material flows, economic savings, or environmental impact was performed in the present study. A limitation of the present study is that avocado seed flour was obtained from a single batch, which may not fully represent the variability associated with different sources or production conditions. In addition, although the present study demonstrated the physicochemical and technological stability of frankfurter-type sausages during refrigerated storage, microbiological safety was not evaluated, and therefore the results should not be interpreted as a comprehensive assessment of product shelf life. Furthermore, the sensory evaluation was conducted using a relatively homogeneous group of young consumers, which may limit the generalizability of the acceptability results to broader population segments. Future research should explore higher incorporation levels, evaluate the effects of avocado seed ingredients on oxidative stability, include microbiological analyses to establish product safety and shelf life under commercial processing and storage conditions, and assess the influence of raw material variability, as well as further investigate the structural interactions between plant-derived fractions and meat protein networks in complex food systems, and determine optimal inclusion levels under different formulation conditions.

## Figures and Tables

**Table 1:** Formulation of frankfurter-type sausages (g/100 g batter) prepared as control (CNF), non-extruded (SNF), and extruded (SEF) avocado seed flour.

Ingredients	Formulation (g/100 g Batter)		
	CNF	SNF	SEF
Turkey meat	68.70	68.70	68.70
Water	21.10	21.10	21.10
NaCl	1.87	1.87	1.87
Smix	0.35	0.35	0.35
Sodium nitrate	0.02	0.02	0.02
Sodium erythorbate	0.05	0.05	0.05
Sodium phosphate	0.40	0.40	0.40
Carmine (E120)	0.01	0.01	0.01
Soy protein concentrate	3.00	3.00	3.00
Modified starch	1.50	1.50	1.50
Corn starch	2.00	2.00	2.00
Soy fiber	1.00	–	–
NEF	–	1.00	–
EF	–	–	1.00

CNF: control formulation containing soy fiber; SNF: formulation containing non-extruded avocado seed flour; SEF: formulation containing extruded avocado seed flour; NEF: non-extruded avocado seed flour; EF: extruded avocado seed flour; Smix: standard flavor mixture (spices, salt, starch, flavorings, and hydrolyzed vegetable protein). Values are expressed as g/100 g of batter. The symbol (–) indicates that the ingredient was not included in the formulation.

**Table 2:** Extraction yields of fractions obtained by enzyme-assisted wet milling from non-extruded (NEF) and extruded (EF) avocado seed flours (% dry basis).

Sample	Fraction 1 (F1)	Fraction 2 (F2)	Fraction 3 (F3)
NEF	40.24 ± 0.67 ^a^	44.73 ± 1.16 ^a^	15.03 ± 0.74 ^b^
EF	30.15 ± 0.89 ^b^	40.25 ± 0.37 ^a^	29.60 ± 0.76 ^a^

Values are expressed as mean ± SD (NEF: *n* = 6; EF: *n* = 4). Within each column, different lowercase letters indicate significant differences between NEF and EF samples (*p* ≤ 0.05). Comparisons were performed using Student’s *t*-test (F1 and F2) or Welch’s *t*-test (F3), depending on variance homogeneity. NEF: non-extruded avocado seed flour; EF: extruded avocado seed flour.

**Table 3:** Chemical composition (% dry basis) of non-extruded (NEF) and extruded (EF) avocado seed flours and their fractions obtained by wet milling.

Parameter	Fraction	NEF	EF
Protein	Whole	4.51 ± 0.11 ^a^	5.23 ± 0.10 ^b^
	F1	4.77 ± 0.11 ^a^	6.01 ± 0.21 ^b^
	F2	4.18 ± 0.01 ^a^	6.19 ± 0.19 ^b^
	F3	3.07 ± 0.12 ^b^	2.37 ± 0.11 ^a^
Fat	Whole	1.18 ± 0.07 ^b^	0.76 ± 0.02 ^a^
	F1	1.50 ± 0.05 ^a^	0.96 ± 0.12 ^a^
	F2	2.57 ± 0.96 ^a^	0.93 ± 0.05 ^a^
	F3	3.48 ± 1.22 ^a^	0.21 ± 0.01 ^a^
Ash	Whole	2.48 ± 0.05 ^a^	2.30 ± 0.10 ^a^
	F1	1.76 ± 0.01 ^b^	1.50 ± 0.05 ^a^
	F2	1.06 ± 0.12 ^a^	1.28 ± 0.09 ^a^
	F3	11.97 ± 0.41 ^a^	24.99 ± 2.46 ^b^
Carbohydrates *	Whole	67.24 ± 0.49 ^a^	67.86 ± 0.25 ^a^
	F1	92.00 ± 0.14 ^a^	91.50 ± 0.29 ^a^
	F2	92.51 ± 1.08 ^a^	91.62 ± 0.15 ^a^
	F3	81.46 ± 2.13 ^a^	73.24 ± 2.79 ^a^
Dietary fiber fractions			
TDF	Whole	24.48 ± 0.45 ^b^	19.83 ± 0.28 ^a^
IDF	Whole	21.17 ± 0.52 ^b^	17.32 ± 0.05 ^a^
SDF	Whole	3.31 ± 0.07 ^b^	2.52 ± 0.24 ^a^

Values are expressed as mean ± SD (*n* = 3). Within each row, different lowercase letters indicate significant differences between NEF and EF samples (*p* ≤ 0.05). Comparisons were performed using Student’s *t*-test or Welch’s *t*-test, depending on variance homogeneity. Dietary fiber fractions were determined only in whole flours. NEF: non-extruded avocado seed flour; EF: extruded avocado seed flour; TDF: total dietary fiber; IDF: insoluble dietary fiber; SDF: soluble dietary fiber. * Carbohydrates were calculated by difference.

**Table 4:** Effect of extrusion on the concentration of individual and total acetogenins in avocado seed flour and their molecular structures (mg/g, dry basis).

Acetogenin	Molecular Structure	Sample		Acetogenin Reduction (%)
		NEF	EF	
AcO-avocadenyne	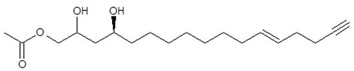	0.79 ± 0.09 ^a^	0.06 ± 0.01 ^b^	91.62
AcO-avocadene	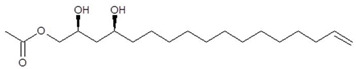	2.94 ± 0.30 ^a^	0.44 ± 0.02 ^b^	84.74
AcO-avocadiene B	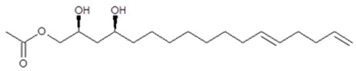	0.28 ± 0.10 ^a^	0.01 ± 0.01 ^b^	93.35
Persediene	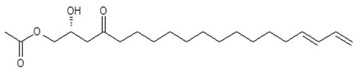	0.24 ± 0.02 ^a^	0.02 ± 0.01 ^b^	91.04
Persenone C	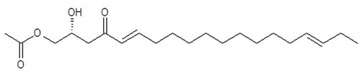	0.73 ± 0.08 ^a^	0.10 ± 0.01 ^b^	85.78
Persenone A	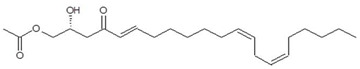	3.11 ± 0.36 ^a^	0.53 ± 0.02 ^b^	82.83
Persin	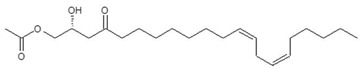	2.56 ± 0.17 ^a^	0.67 ± 0.02 ^b^	73.74
Persenone B	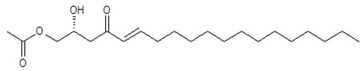	1.32 ± 0.22 ^a^	0.20 ± 0.01 ^b^	84.89
Total acetogenins		11.99 ± 1.37 ^a^	2.07 ± 0.07 ^b^	82.77

Values are expressed as mean ± SD (*n* = 3). Within each row, different lowercase letters indicate significant differences between NEF and EF samples (*p* ≤ 0.05). Comparisons were performed using Student’s *t*-test or Welch’s *t*-test, depending on variance homogeneity. NEF: non-extruded avocado seed flour; EF: extruded avocado seed flour. Molecular structures were drawn based on the structural characterization reported by Rodríguez-Sánchez [20].

**Table 5:** Antioxidant activity and techno-functional properties of non-extruded (NEF) and extruded (EF) avocado seed flours and their fractions obtained by wet milling (dry basis).

Parameter	Fraction	NEF	EF
AA (%)	Whole	53.34 ± 1.63 ^b^	19.95 ± 3.96 ^a^
	F1	35.32 ± 1.95 ^a^	27.47 ± 7.62 ^a^
	F2	30.30 ± 4.52 ^a^	11.58 ± 1.02 ^a^
	F3	23.90 ± 8.99 ^a^	7.34 ± 2.37 ^a^
WAI (g/g)	Whole	2.87 ± 0.01 ^a^	3.91 ± 0.06 ^b^
	F1	3.57 ± 0.04 ^a^	3.99 ± 0.07 ^b^
	F2	2.55 ± 0.01 ^a^	3.79 ± 0.01 ^b^
	F3	0.50 ± 0.04 ^a^	1.27 ± 0.06 ^b^
OAI (g/g)	Whole	2.12 ± 0.01 ^b^	1.84 ± 0.01 ^a^
	F1	2.31 ± 0.06 ^b^	1.96 ± 0.05 ^a^
	F2	2.16 ± 0.03 ^b^	1.81 ± 0.02 ^a^
	F3	1.95 ± 0.03 ^a^	1.82 ± 0.03 ^a^
EC (%)	Whole	43.86 ± 0.57 ^a^	42.97 ± 0.69 ^a^
	F1	42.45 ± 0.40 ^a^	44.81 ± 0.32 ^b^
	F2	41.88 ± 0.74 ^a^	43.88 ± 0.73 ^a^
	F3	45.45 ± 0.39 ^a^	44.40 ± 1.03 ^a^
ES (%)	Whole	42.67 ± 0.60 ^a^	42.97 ± 0.69 ^a^
	F1	40.81 ± 1.13 ^a^	43.57 ± 0.31 ^a^
	F2	41.88 ± 0.74 ^a^	43.46 ± 1.46 ^a^
	F3	42.97 ± 0.41 ^a^	43.57 ± 0.31 ^a^

Values are expressed as mean ± SD (*n* = 3). Within each row, different lowercase letters indicate significant differences between NEF and EF samples (*p* ≤ 0.05). Comparisons were performed using *t*-tests with adjustment for variance heterogeneity when required. NEF: non-extruded avocado seed flour; EF: extruded avocado seed flour; AA: antioxidant activity; WAI: water absorption index; OAI: oil absorption index; EC: emulsifying capacity; ES: emulsion stability.

**Table 6:** Rapid Visco Analyzer (RVA) pasting properties of non-extruded avocado seed flour (NEF), fractions obtained by enzyme-assisted wet milling (NEF F1, NEF F2) and extruded avocado seed flour (EF).

Ingredient	Parameters			
	PV (cP)	TV (cP)	BD (cP)	FV (cP)
NEF	1171 ± 4 ^b^	1136 ± 5 ^b^	35 ± 1 ^b^	2350 ± 27 ^b^
NEF F1	1160 ± 73 ^b^	1139 ± 81 ^b^	21 ± 8 ^c^	1958 ± 100 ^b^
NEF F2	3881 ± 22 ^a^	2670 ± 17 ^a^	1210 ± 4 ^a^	4509 ± 429 ^a^
EF	138 ± 2 ^c^	133 ± 1 ^c^	5 ± 1 ^d^	193 ± 5 ^c^
	SB (cP)	Peak Time (min)	Pasting Temp (°C)	
NEF	1214 ± 22 ^b^	5.90 ± 0.17 ^b^	81.97 ± 0.03 ^b^	
NEF F1	818 ± 18 ^b^	6.70 ± 0.23 ^a^	86.37 ± 0.48 ^a^	
NEF F2	1838 ± 446 ^a^	5.23 ± 0.04 ^c^	82.08 ± 0.08 ^c^	
EF	60 ± 4 ^c^	6.94 ± 0.06 ^a^	—	

Values are expressed as mean ± SD (*n* = 3). Within each column, different lowercase letters indicate significant differences among samples (*p* ≤ 0.05). Data were analyzed using one-way ANOVA followed by Tukey’s honestly significant difference (HSD) test. NEF: non-extruded avocado seed flour; F1 and F2: fractions obtained from non-extruded flour by enzyme-assisted wet milling; EF: extruded avocado seed flour; PV: peak viscosity; TV: trough viscosity; BD: breakdown (PV − TV); FV: final viscosity; SB: setback (FV − TV). The pasting temperature for EF could not be determined (—).

**Table 7:** In vitro and relative protein digestibility (%) of non-extruded (NEF) and extruded (EF) avocado seed flours and their fractions obtained by enzyme-assisted wet milling.

Fraction	NEF	EF
In vitro protein digestibility (%)		
Whole	76.64 ± 0.21 ^b^	72.90 ± 0.48 ^a^
F1	72.78 ± 0.10 ^a^	75.19 ± 0.21 ^b^
F2	75.49 ± 0.75 ^b^	71.21 ± 0.21 ^a^
F3	70.67 ± 0.55 ^a^	70.25 ± 0.28 ^a^
Relative protein digestibility (%)		
Whole	58.15 ± 1.10 ^b^	41.53 ± 1.99 ^a^
F1	38.98 ± 2.41 ^a^	51.76 ± 0.96 ^b^
F2	53.36 ± 4.43 ^b^	30.99 ± 1.11 ^a^
F3	26.84 ± 1.92 ^a^	25.56 ± 2.00 ^a^

Values are expressed as mean SD (*n* = 3). Within each row, different lowercase letters indicate significant differences between NEF and EF samples (*p* ≤ 0.05). Comparisons were performed using *t*-tests with adjustment for variance heterogeneity when required. NEF: non-extruded avocado seed flour; EF: extruded avocado seed flour; F1–F3: enzyme-assisted wet-milling fractions.

**Table 8:** Chemical composition (%) and energy value (kcal/62.5 g portion) of CNF, SNF, and SEF frankfurters at 0 and 35 days of refrigerated storage (4 ± 1 °C).

	Time (d)	Sample		
		CNF	SNF	SEF
Chemical composition				
Moisture	0	65.96 ± 0.15 ^a^	65.48 ± 0.15 ^a^	65.53 ± 0.61 ^a^
	35	65.51 ± 0.15 ^a^	65.29 ± 0.22 ^a^	65.31 ± 0.14 ^a^
Protein	0	12.71 ± 0.30 ^a^	12.55 ± 0.68 ^a^	12.19 ± 0.51 ^a^
	35	12.77 ± 0.11 ^a^	12.49 ± 0.12 ^a^	12.25 ± 0.29 ^a^
Fat	0	12.96 ± 0.13 ^aA^	12.50 ± 0.15 ^bA^	12.63 ± 0.04 ^bA^
	35	12.60 ± 0.25 ^aB^	12.28 ± 0.19 ^bB^	11.92 ± 0.22 ^bB^
Ash	0	1.22 ± 0.06 ^abA^	1.13 ± 0.07 ^bA^	1.31 ± 0.06 ^aA^
	35	0.91 ± 0.04 ^abB^	0.88 ± 0.09 ^bB^	0.97 ± 0.08 ^aB^
Carbohydrates	0	7.16 ± 0.55 ^bB^	8.34 ± 0.80 ^aB^	8.34 ± 1.17 ^aB^
	35	8.21 ± 0.08 ^bA^	9.05 ± 0.07 ^aA^	9.54 ± 0.48 ^aA^
Dietary fiber fractions				
TDF	0	7.01 ± 0.01 ^a^	5.83 ± 0.29 ^a^	5.75 ± 0.78 ^a^
	35	5.73 ± 0.71 ^a^	5.52 ± 0.85 ^a^	4.75 ± 0.78 ^a^
IDF	0	5.25 ± 0.11 ^a^	3.95 ± 0.25 ^a^	4.45 ± 0.37 ^a^
	35	4.54 ± 0.14 ^a^	4.54 ± 0.28 ^a^	3.38 ± 0.92 ^a^
SDF	0	1.76 ± 0.11 ^a^	1.88 ± 0.04 ^a^	1.31 ± 0.40 ^a^
	35	1.19 ± 0.57 ^a^	0.98 ± 0.57 ^a^	1.37 ± 0.13 ^a^
Energy value (kcal/62.5 g)	0	122.53 ± 0.23 ^a^	122.55 ± 0.42 ^a^	122.36 ± 1.53 ^a^
	35	123.34 ± 1.05 ^a^	122.94 ± 0.94 ^a^	121.55 ± 0.99 ^a^
Significance		T	S	T × S
Moisture		NS	NS	NS
Protein		NS	NS	NS
Fat		***	***	NS
Ash		*	***	NS
Carbohydrates		*	**	NS
TDF		NS	NS	NS
IDF		NS	NS	NS
SDF		NS	NS	NS
Energy value		NS	NS	NS

Values are expressed as mean ± SD (*n* = 3). Within each row, different lowercase letters indicate significant differences among treatments (CNF, SNF, SEF) at the same storage time (*p* ≤ 0.05), while different uppercase letters within the same column indicate significant differences between storage times (0 and 35 days) for the same treatment (*p* ≤ 0.05). Data were analyzed using a two-way ANOVA within a General Linear Model (GLM), considering treatment (T) and storage time (S) as fixed factors, followed by Tukey’s honestly significant difference (HSD) test when significant effects were detected. The significance of main effects is presented as T (treatment), S (storage time), and T × S (interaction). * *p* < 0.05; ** *p* < 0.01; *** *p* < 0.001; NS, not significant. CNF: control frankfurter formulated with soy fiber; SNF: frankfurter formulated with non-extruded avocado seed flour; SEF: frankfurter formulated with extruded avocado seed flour; TDF: total dietary fiber; IDF: insoluble dietary fiber; SDF: soluble dietary fiber.

**Table 9:** Instrumental color parameters (CIE L*a*b*, chroma C*, and hue angle h°) of CNF, SNF, and SEF frankfurters during refrigerated storage (4 °C).

Color Parameters	Time (d)	Sample		
		CNF	SNF	SEF
L*				
	0	66.48 ± 1.26 ^a^	57.91 ± 1.81 ^b^	57.60 ± 2.09 ^b^
	7	65.89 ± 2.24 ^a^	61.64 ± 2.34 ^b^	57.84 ± 1.75 ^c^
	14	66.43 ± 0.89 ^a^	60.29 ± 2.35 ^b^	57.83 ± 2.04 ^c^
	21	64.64 ± 2.69 ^a^	60.74 ± 2.62 ^b^	60.26 ± 2.22 ^b^
	28	64.43 ± 2.57 ^a^	57.63 ± 2.16 ^b^	57.19 ± 2.11 ^b^
	35	62.85 ± 1.4 ^a^	60.49 ± 2.10 ^b^	60.10 ± 2.09 ^b^
a*				
	0	11.74 ± 0.91 ^b^	15.69 ± 1.10 ^a^	14.55 ± 1.54 ^a^
	7	12.09 ± 1.46 ^b^	13.23 ± 1.77 ^ab^	14.62 ± 1.03 ^a^
	14	11.67 ± 0.27 ^b^	14.24 ± 1.56 ^a^	14.85 ± 0.92 ^a^
	21	13.13 ± 1.48 ^a^	14.43 ± 2.26 ^a^	13.69 ± 1.41 ^a^
	28	12.74 ± 1.58 ^b^	16.16 ± 1.32 ^a^	14.96 ± 1.22 ^a^
	35	13.95 ± 0.77 ^a^	14.25 ± 1.62 ^a^	13.01 ± 1.08 ^a^
b*				
	0	15.64 ± 0.50 ^c^	20.11 ± 1.31 ^a^	18.69 ± 0.85 ^b^
	7	17.00 ± 1.08 ^b^	18.07 ± 1.38 ^ab^	18.54 ± 1.29 ^a^
	14	16.16 ± 0.69 ^c^	20.37 ± 1.24 ^a^	18.98 ± 0.70 ^b^
	21	17.06 ± 1.52 ^a^	19.00 ± 1.83 ^a^	18.58 ± 1.71 ^a^
	28	17.28 ± 1.17 ^b^	20.62 ± 1.03 ^a^	19.49 ± 1.16 ^a^
	35	18.04 ± 0.76 ^b^	19.66 ± 1.23 ^a^	17.92 ± 1.12 ^b^
Chroma (C*)				
	0	19.57 ± 0.81 ^c^	25.51 ± 1.61 ^a^	23.69 ± 1.57 ^b^
	7	20.88 ± 1.64 ^b^	22.41 ± 2.07 ^ab^	23.61 ± 1.59 ^a^
	14	19.94 ± 0.63 ^b^	24.88 ± 1.55 ^a^	24.10 ± 1.08 ^a^
	21	21.53 ± 2.10 ^a^	23.87 ± 2.80 ^a^	23.09 ± 2.12 ^a^
	28	21.48 ± 1.84 ^b^	26.20 ± 1.55 ^a^	24.58 ± 1.61 ^a^
	35	22.80 ± 1.04 ^ab^	24.30 ± 1.82 ^a^	22.15 ± 1.45 ^b^
Hue angle (h°)				
	0	53.16 ± 1.91 ^a^	52.03 ± 1.28 ^a^	52.20 ± 1.94 ^a^
	7	54.68 ± 2.11 ^a^	53.91 ± 2.22 ^ab^	51.73 ± 0.99 ^b^
	14	54.13 ± 1.13 ^ab^	55.10 ± 2.84 ^a^	51.98 ± 0.97 ^b^
	21	52.48 ± 0.85 ^a^	52.95 ± 1.87 ^a^	53.64 ± 1.58 ^a^
	28	53.70 ± 1.79 ^a^	51.96 ± 1.39 ^b^	52.53 ± 1.14 ^ab^
	35	52.30 ± 0.78 ^a^	54.13 ± 2.16 ^a^	54.04 ± 1.46 ^a^
Significance		T	S	T × S
L*		***	**	***
a*		***	*	***
b*		***	**	***
C*		***	**	***
h°		*	*	***

Values are expressed as mean ± SD (*n* = 9 measurements per treatment and storage time). Within each storage time, different lowercase letters indicate significant differences among treatments (CNF, SNF, SEF) (*p* ≤ 0.05). Data were analyzed using a two-way ANOVA within a General Linear Model (GLM), considering treatment (T) and storage time (S) as fixed factors, followed by Tukey’s honestly significant difference (HSD) test for mean comparisons at each storage time when significant effects were detected. The significance of main effects is presented as T (treatment), S (storage time), and T × S (interaction). * *p* < 0.05; ** *p* < 0.01; *** *p* < 0.001. CNF: control frankfurter formulated with soy fiber; SNF: frankfurter formulated with non-extruded avocado seed flour; SEF: frankfurter formulated with extruded avocado seed flour.

**Table 10:** Physicochemical (pH, cooking loss, and water activity) and textural (hardness) properties of CNF, SNF, and SEF frankfurters during refrigerated storage (4 ± 1 °C).

Parameters	Time (d)	Sample		
		CNF	SNF	SEF
pH				
	0	6.26 ± 0.04 ^b^	6.52 ± 0.02 ^a^	6.13 ± 0.01 ^c^
	7	6.26 ± 0.01 ^b^	6.56 ± 0.04 ^a^	6.16 ± 0.01 ^c^
	14	6.27 ± 0.02 ^b^	6.39 ± 0.01 ^a^	6.21 ± 0.02 ^c^
	21	6.30 ± 0.03 ^b^	6.44 ± 0.03 ^a^	6.24 ± 0.02 ^c^
	28	6.33 ± 0.01 ^a^	6.36 ± 0.04 ^a^	6.23 ± 0.02 ^b^
	35	6.31 ± 0.01 ^a^	6.30 ± 0.02 ^a^	6.24 ± 0.01 ^b^
Water activity (Aw)				
	0	0.987 ± 0.008	0.985 ± 0.012	0.994 ± 0.003
	7	0.988 ± 0.008	0.990 ± 0.006	0.987 ± 0.002
	14	0.984 ± 0.005	0.986 ± 0.004	0.988 ± 0.005
	21	0.985 ± 0.008	0.986 ± 0.009	0.984 ± 0.003
	28	0.979 ± 0.005	0.987 ± 0.006	0.982 ± 0.003
	35	0.986 ± 0.006	0.990 ± 0.002	0.985 ± 0.004
Cooking loss (%)				
	0	3.77 ± 0.19 ^a^	2.44 ± 0.26 ^b^	3.20 ± 0.56 ^ab^
	7	3.28 ± 0.11 ^a^	2.46 ± 0.20 ^b^	2.56 ± 0.20 ^b^
	14	3.30 ± 0.24 ^a^	2.49 ± 0.15 ^b^	2.79 ± 0.16 ^b^
	21	3.26 ± 0.30 ^a^	2.43 ± 0.28 ^b^	2.97 ± 0.14 ^ab^
	28	2.08 ± 0.22 ^a^	2.29 ± 0.37 ^a^	1.67 ± 0.17 ^ab^
	35	1.92 ± 0.10 ^a^	1.99 ± 0.07 ^a^	2.06 ± 0.22 ^ab^
Hardness (N)				
	0	1.19 ± 0.09 ^a^	1.17 ± 0.07 ^a^	1.23 ± 0.08 ^a^
	7	1.15 ± 0.05 ^b^	1.21 ± 0.12 ^ab^	1.26 ± 0.10 ^a^
	14	1.09 ± 0.09 ^b^	1.17 ± 0.09 ^ab^	1.23 ± 0.08 ^a^
	21	1.16 ± 0.07 ^b^	1.40 ± 0.08 ^a^	1.41 ± 0.09 ^a^
	28	1.01 ± 0.19 ^b^	1.27 ± 0.12 ^a^	1.29 ± 0.07 ^a^
Significance		T	S	T × S
pH		***	***	***
Cooking loss		***	***	***
Aw		NS	NS	NS
Hardness		***	***	***

Values are expressed as mean ± SD. For pH, cooking loss, and water activity, *n* = 3 samples per treatment and storage time; for hardness, *n* = 7–12 measurements. Within each storage time, different lowercase letters indicate significant differences among treatments (CNF, SNF, SEF) (*p* ≤ 0.05). Data were analyzed using a two-way ANOVA within a General Linear Model (GLM), considering treatment (T) and storage time (S) as fixed factors, followed by Tukey’s honestly significant difference (HSD) test for mean comparisons at each storage time when significant effects were detected. The significance of main effects is presented as T (treatment), S (storage time), and T × S (interaction). *** *p* < 0.001; NS, not significant. CNF: control frankfurter formulated with soy fiber; SNF: frankfurter formulated with non-extruded avocado seed flour; SEF: frankfurter formulated with extruded avocado seed flour.

**Table 11:** Consumer sensory attributes of frankfurters formulated with commercial soy fiber (CNF), non-extruded avocado seed flour (SNF), or extruded avocado seed flour (SEF), expressed as median (Q1–Q3).

	Samples					
Parameters	CNF		SNF		SEF	
Flavor	4.00	(4.00–7.00) ^b^	5.00	(5.00–8.00) ^a^	5.00	(5.00–8.00) ^a^
Texture	7.00	(5.00–8.00) ^ns^	7.00	(5.00–8.00) ^ns^	7.00	(5.00–8.00) ^ns^
Freshness	7.00	(6.00–8.00) ^ns^	7.00	(6.00–8.00) ^ns^	7.00	(6.00–8.00) ^ns^
Overall acceptability	6.00	(5.00–7.00) ^b^	7.00	(6.00–8.00) ^ab^	7.00	(6.00–8.00) ^a^

Values are expressed as median (Q1–Q3) (*n* = 106 consumers per sample). Differences among samples were analyzed using the Kruskal–Wallis test, followed by pairwise Mann–Whitney U tests with Bonferroni correction (adjusted α = 0.0167). Different lowercase letters within the same row indicate significant differences among samples (*p* ≤ 0.0167), while rows without letters indicate no significant differences. CNF: control frankfurter formulated with soy fiber; SNF: frankfurter formulated with non-extruded avocado seed flour; SEF: frankfurter formulated with extruded avocado seed flour.

## Data Availability

The original contributions presented in this study are included in the article. Further inquiries can be directed to the corresponding authors.
